# Sexual and gender minority parents’ experiences of lactation: a meta-ethnographic review

**DOI:** 10.1186/s13006-026-00897-8

**Published:** 2026-09-21

**Authors:** Malena Chronholm Bøyesen, Karolina Linden, Lisa Goldkuhl

**Affiliations:** 1 Institute of Health and Care Sciences, Sahlgrenska Academy, University of Gothenburg, Arvid Wallgrens Backe, Box 457, Gothenburg, 405 30 Sweden; 2 Department of Obstetrics and Gynaecology, Sahlgrenska University Hospital, Region Västra Götaland, Gothenburg, Sweden

**Keywords:** Sexual and gender minorities, Lactation, Parenting, Queer theory, Intersectional framework, Meta-ethnography, Systematic literature review

## Abstract

**Background:**

Although lactation has been widely studied, existing evidence largely reflects heterosexual and cisgender parents’ experiences. Sexual and gender minority (SGM) parents may face distinct embodied, social, and healthcare-related factors that shape lactation and access to support. This study aimed to synthesise and interpret existing qualitative research on SGM parents’ experiences of lactation.

**Methods:**

A systematic literature review following a meta-ethnographic method developed by Noblit and Hare was conducted. The analysis involved a synthesis informed by queer theory and an intersectional perspective.

**Results:**

Of the 528 screened records, 16 qualitative studies were included in the review. The analysis resulted in the core theme; *Normative assumptions as barriers to lactation*, which describes the main obstacles for a satisfactory lactation experience for SGM parents. The following four subordinate themes were identified: *Layers of vulnerability and resilience*; *Negotiating identity through lactation*; *Creating connection through lactation* and; *The importance of support during lactation*.

**Conclusions:**

Lactation is commonly framed by normative assumptions about heterosexuality, cisgender identity, womanhood, and motherhood. SGM parents may not conform to one or more of these assumptions, which can create barriers to positive lactation and infant-feeding experiences. These findings highlight the need for inclusive and affirming lactation care, as well as intersectional research on how identity markers shape SGM parents’ experiences. Prejudice, cis- and heteronormative assumptions, and limited professional knowledge indicate important gaps in current healthcare practice.

**Supplementary Information:**

The online version contains supplementary material available at https://doi.org/10.1186/s13006-026-00897-8.

## Background

Over the last few decades, increasing attention has focused on the benefits of lactation. Breastfeeding has been associated with parent–infant attachment, with longer durations of lactation associated with higher levels of attachment security [1]. Induced lactation has also been described by non-gestational parents as meaningful [2], enabling them to establish a milk supply without having been pregnant [3]. Human milk contains antibodies that contribute to infant immune protection and development [4]. Lactation and nursing have also been associated with long-term health benefits for the lactating parent, including reduced risks of breast [5, 6], ovarian [5] and endometrial cancer [7], cardiovascular disease [8, 9], type 2 diabetes [10] and postpartum depression [11].

Sexual and gender minority (SGM) is an umbrella term used to describe diverse sexual orientations, gender identities and expressions, and sex characteristics, including, but not limited to, people who identify as lesbian, gay, bisexual, asexual, transgender, Two Spirit, queer, and/or intersex [12]. SGM couples who are expecting a child may encounter inadequate care, misunderstanding and stigma within the healthcare sector [13]. Individualised and person-centred care may reduce the risk of insensitive or inappropriate care [14]. At present, the World Professional Association for Transgender Health (WPATH) Standards of Care provide no recommendations or guidelines for counselling about breastfeeding before surgery [15]. Preoperative counselling may be important because surgical procedures can affect future breastfeeding, particularly in the event of complications such as nipple necrosis [16]. The limited availability, implementation, and specificity of clinical guidance regarding lactation means that the advice given by healthcare professionals may vary. There is a risk that SGM parents seeking care are not met with the support and guidance they need or ask for [17], which puts SGM parents at an even greater risk of experiencing harm. To our knowledge, no review has synthesised qualitative evidence specifically on SGM parents’ experiences of lactation. Existing syntheses have addressed related reproductive experiences, for example a meta-ethnography on transgender men’s experiences of conception, pregnancy, and childbirth [18], but did not focus on lactation. Moreover, qualitative studies on SGM parents and lactation are fragmented, often examining specific subgroups (e.g. transgender and non-binary parents or same-sex couples) in isolation, which limits a broader understanding of shared and divergent experiences across SGM populations. A synthesis is needed to identify the factors SGM parents perceive as most important for positive lactation experiences and to inform inclusive, person-centred lactation support. Therefore, this study aimed to synthesise and interpret existing qualitative research on SGM parents’ experiences of lactation.

## Method

### Study design

A systematic literature review following the meta-ethnographic method developed by Noblit and Hare [19] was used. This approach is well suited for understanding how SGM parents’ lactation experiences are represented in the qualitative literature. By identifying recurring themes and patterns across personal narratives, it supports an in-depth analysis of complex social phenomena and enables the development of new, integrative interpretations. This study adhered to the eMERGE reporting guidance [20] for meta-ethnographic methods (Supplementary file 1).

### Theoretical framework

In the interpretation of the findings, we draw on queer theory [21] and intersectionality [22]. These perspectives were chosen because they emphasise the forces of identity, power, and social norms that shape individual experiences, and offer conceptual tools to explore how such dynamics influence SGM parents’ experiences of lactation. As with any human experience, SGM parents’ experiences of lactation are shaped by the cultural contexts in which they occur. This means that unwritten rules and expectations about what is considered normative, in this case heterosexuality and cisgender identity, may lead to prejudiced assumptions about those who do not conform to societal norms. Queer Theory challenges the notion of gender norms and, in doing so, encourages alternative ways of understanding lived experiences [21].

We conceptualised cisnormativity as the assumption that gender identity corresponds with sex assigned at birth, and heteronormativity as the privileging of heterosexuality and heterosexual family structures as normative. The understanding of these concepts as social and institutional constructs shapes the experiences of lactation, particularly for SGM parents. The concept of cis- and heteronormativity highlight that the experiences are situated and understood within these constructs, rather than interpreted solely as individual experiences.

Although a queer theoretical perspective critically challenges normative assumptions about gender and sexuality, it overlooks the existing differences and contradictions within sexual minority groups [23], overlooking the fact that any group is composed of individuals who each embody a unique constellation of privileges and vulnerabilities. Vulnerability can be understood as existing along a continuum of severity and as arising from specific situations and contexts [24]. Viewed through an intersectional lens, vulnerability should not be conceptualised as an inherent characteristic of individuals, but rather as relationally produced through encounters with healthcare practices, social norms, power structures, and broader contextual conditions. An intersectional perspective [22] avoids generalisation and addresses the interplay of social identities and health outcomes [25] concerning SGM parents’ experiences of lactation. Intersectional theory analyses individuals based on the intersections of social categories, such as sexual orientation, gender identity or the parental role. The focus is based on how these categories cooperate and affect the needs and experiences of the individual.

### Data collection

The search strategy was developed using the Population, Exposure and Outcome (PEO) framework, which also guided the formulation of the inclusion criteria for the review. Search terms were defined in collaboration with university librarians at the Gothenburg University Medical Library. Broad definitions of lactation and Sexual and Gender Minority were applied to capture the widest possible range of relevant findings (Supplementary file 2). Eligible studies included qualitative, peer-reviewed, full-text articles published in English or Scandinavian languages that focused on SGM parents with lived experience of lactation. No publication date limits were applied. This decision was informed by the scarcity of research on the topic, and the potential value of examining whether shifting gender norms over time may have influenced SGM parents’ experiences.

**Fig. 1:**
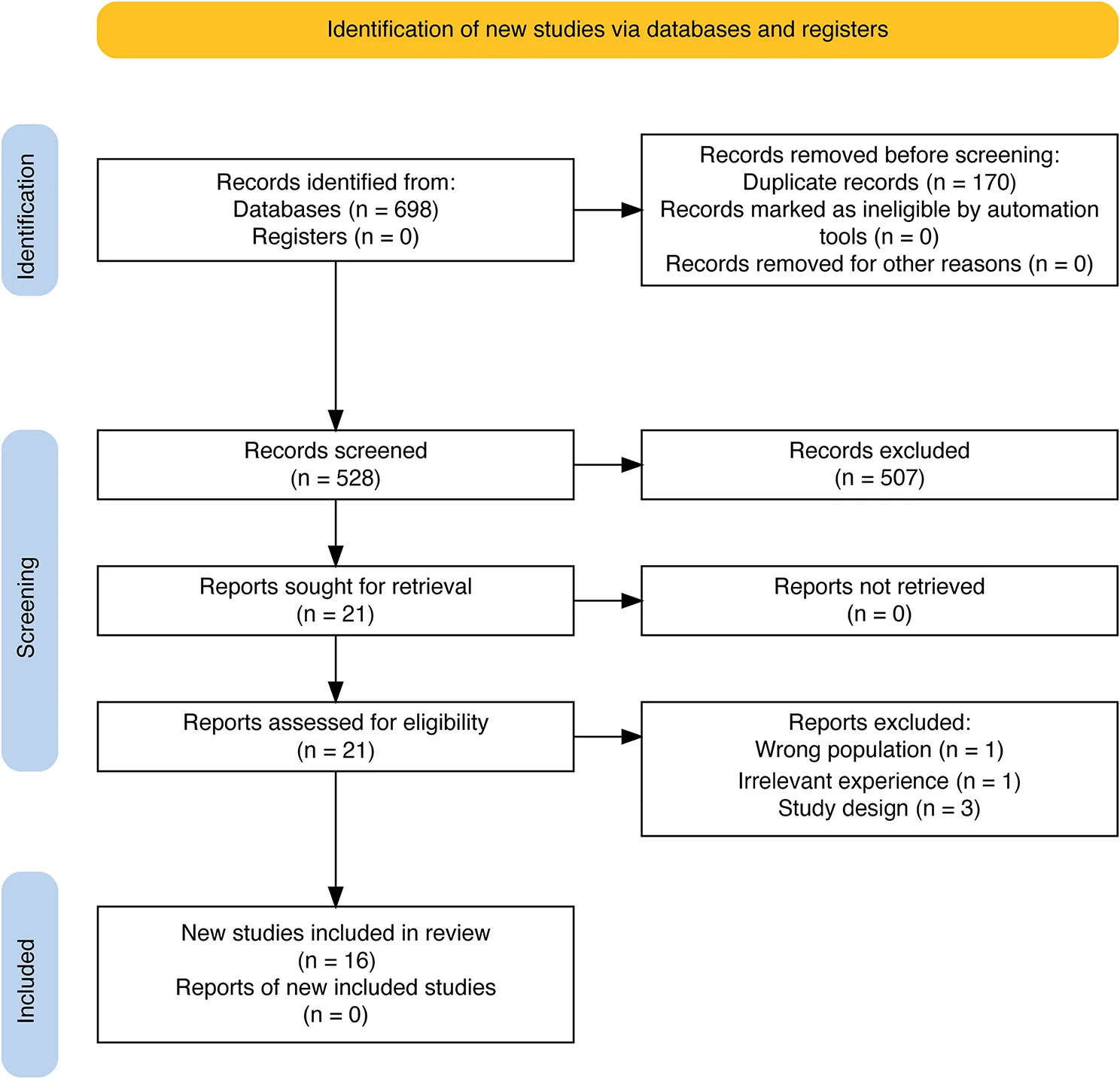
PRISMA-flow chart [31], illustration of the selection process. After duplicate removal, a total of 528 articles were identified. After screening and full text review, 16 articles were included in the final analysis

Database searches were conducted in PubMed, CINAHL and Scopus, selected for their relevance to health, social science and interdisciplinary research. Duplicates were removed both manually and with automated functions in the databases. The filtering process used in this study is described in a flowchart (Fig. 1) according to the Preferred Reporting Items for Systematic Reviews and Meta-Analyses [26]. A structured literature search was conducted in February 2025, updated in October 2025, and repeated on 20 August 2026 using the same search strategy. The search identified 698 records, of which 528 remained after duplicate removal. All articles were screened by MCB and LG against the predefined eligibility criteria. Any disagreements were resolved through discussion among all authors. Articles identified by either reviewer as potentially relevant proceeded to full-text assessment.

In total, 21 articles were assessed for eligibility. Five articles were excluded after full-text assessment: three due to study design, one due to lack of relevant experiences and one because it did not address SGM parents (Supplementary file 3). Consequently, 16 articles met the inclusion criteria and were included in the review (Table 1).

All included articles were quality appraised (Supplementary file 4). To assess the scientific quality and relevance of the selected articles, the Critical Appraisal Skills Programme (CASP) criteria [27] was applied, specifically using the Qualitative Studies Checklist. The tool assesses methodological rigour across ten predefined criteria and categorises each study as *methodologically sound*, *relatively poor methodology*, or *uncertain*. This ensured that the studies included in the review demonstrated sufficient quality and reliability. Three studies [28–30] lacked formal ethical approval but demonstrated clear ethical considerations, which supported their inclusion. Key data were extracted into the CASP’s predesigned template.

### Data analysis

The analysis followed the seven steps of meta-ethnography, which are designed to synthesise findings from multiple qualitative studies to generate new interpretations [19]. This process ensures that the translation remains faithful to the content of the original studies while also integrating and comparing their contributions into a broader and more coherent synthesis. The seven phases involve identifying the area of interest, deciding what is relevant, reading and rereading the studies, deciding how the chosen studies are related, translating the studies in relation to one another, synthesising translations, and presenting the synthesis.

Based on an initial comparison of the included studies, a list of non-abstract key phrases was developed (Supplementary file 5). These were subsequently interpreted and reinterpreted, allowing the studies to be translated into one another and placed in dialogue. Throughout this process, differences in analytical approaches across the included studies were considered when comparing and translating concepts, with attention to the context and level of interpretation within each study. In this way, the material was given new meaning within a coherent and integrated whole [19].

The research question focuses on challenging normative assumptions, such as the idea that parents who lactate are homogenic, heterosexual and cisgender. The queer theoretical perspective [21] helps understanding socially constructed ideas of parenting and lactation, and emphasises the importance of studies that reflect parental lactational experiences beyond traditional gender norms. As registered midwives with expertise in a variety of fields beyond reproductive and perineal health, such as specialist nursing in psychiatric care and academic training in gender studies, we bring a multidimensional perspective to this study. This may influence both data collection and analysis. We have aimed to maintain a critical approach throughout the research process through ongoing discussion and reflection regarding our perspectives, assumptions, and prior knowledge, and how these factors may influence our analysis.

## Results

A total of 16 articles were included in the final review (Table 1). The studies were conducted in Brazil (*n* = 3), Canada (*n* = 3), Spain (*n* = 1), Sweden (*n* = 3), The Netherlands (*n* = 1), United Kingdom (*n* = 1) and in the USA (*n* = 4). Overall, these studies represent the views of 257 SGM parents, a group that consists of female same-sex couples, transmasculine, transfeminine and nonbinary individuals. The included studies were conducted between 2008 and 2026. Data was primarily collected through qualitative interviews (*n* = 14), including in-depth, semi-structured (*n* = 8), and reflective formats. Written responses from questionnaires were also analysed (*n* = 1). The studies used a variety of analytical approaches, including inductive (*n* = 3) methods, and were grounded in thematic analysis (*n* = 5), grounded theory (*n* = 3), and interpretive description (*n* = 1). One study applied a mixed methods design [32], and another was conducted as a pilot study [33]. Since the meta-ethnography is based on qualitative findings, the qualitative component of the mixed methods study was assessed using the CASP checklist, rather than assessing the overall study design. 

**Table 1:** Included studies

Authors (Year), country	Study design	Study aim	Data collection	Participants
Cazorla-Ortiz G, Galbany-Estragués P, Obregón-Gutiérrez N, Goberna-Tricas J. (2020), Spain [28]	A qualitative design with a deductive thematic analysis.	To describe and interpret the challenges faced by mothers who undergo induced breastfeeding and relactation for adopted infants, infants born via surrogacy, and infants born to same-sex female partners.	Semi-structured interviews with open-ended questions.	9 women (3 same-sex couples)
Falck FAOK, Dhejne CMU, Frisén LMM, Armuand GM. (2024), Sweden [34]	A qualitative design, with a thematic content analysis.	To examine how gestational gender-diverse individuals in Sweden experience the process of planning and undergoing pregnancy, delivery, and nursing.	Individual interviews	12 transgender men/non-binary individuals
Falck, F., Frisén, L., Dhejne, C., & Armuand, G. (2021), Sweden [35]	A qualitative design, with a thematic content analysis.	To investigate how trans masculine individuals experience healthcare encounters in connection with pregnancy, delivery and nursing, in a setting where mandatory sterilisation to change legal gender was recently removed.	Individual interviews	12 transgender men/non-binary individuals
Galvão DLS, Araújo WJS, Brandão Neto W, Barros MBSC, Monteiro EMM. (2024), Brazil [36]	An exploratory, descriptive qualitative study, with a thematic analysis.	To, from an intersectional standpoint, grasp the challenges experienced by health professionals and service users of human milk banks in provision of care for transgender men chestfeeding.	Individual interviews	2 bisexual transmen
Goldberg, A. E., Downing, J. B., & Sauck, C. C. (2008), USA 27/08/2026 13:54:00 [29]	A qualitative study with a grounded theory approach.	To explore how lesbian mothers perceive their 3 1/2-year-old children’s parental preferences in families in which one mother is genetically linked to the child.	Individual interviews and questionnaires	30 same-sex couples − 60 women
Hoffkling A, Obedin-Maliver J, Sevelius J. (2017), USA [30]	A qualitative study with a grounded theory approach.	To understand the needs of transgender men who had given birth.	Individual interviews	10 transgender men
Jackson, J. E., Wild, R., Hallam, J., Graves, R., Woodstein, B. J., & Stothard, P. (2024), United Kingdom [37]	A qualitative study using a reflexive thematic analysis.	To contribute to the recent body of literature exploring the needs of trans and non-binary people during early parenting as a continued effort for addressing health inequity.	Digital individual semi-structured interviews	7 trans or non-binary participants
Juntereal NA, Spatz DL. (2020), USA [32]	A mixed methods design. The qualitative part is analysed using content analysis.	To explore the lactation experience and level of lactation support of birth mothers in a same-sex (two female) relationship.	Individual interviews	18 gestational mothers
Klittmark S, Niit JKP, Nerström E, Grundström H, Nieminen K, Wells MB, Malmquist A. (2024), Sweden [38]	A qualitative interview methodology using a thematic analysis.	To explore reproductive injustices in postpartum care for LBTQ people.	Qualitative cross-sectional interview methodology.	22 LBTQ birth and non-birth parents
MacDonald T, Noel-Weiss J, West D, Walks M, Biener M, Kibbe A, Myler E. (2016), Canada [39]	A qualitative design, using interpretive description methodology.	To answer an open research question, “What are the experiences of transmasculine individuals with pregnancy, birthing, and feeding their newborns?”	Questionnaires and individual, semi-structured interviews	22 participants who self-identified as transmasculine
Melo, F. E. B. and F. B. Scagliusi (2026), Brazil [40]	Qualitative research study	To narrate and analyze how couples of cisgender women perceive and give meaning to the experience of double breastfeeding, with the analysis based on feminist authors from the Global South, in line with a process of constant positionality, reflecting the negotiations that occur in the fieldand an understanding of the complexity of the ethical commitment of social research with vulnerable participants.	To begin the analysis, a broad research question was proposed: What is the experience of following lactation induction protocols for cisgender women who opted for double breastfeeding? To answer this question, a thematic content analysis was used.	Seven cisgender white women who have relationships with women (*n* = 7)
Pelka S. (2009), USA [41]	A qualitative, ethnographic design.	How variables common to lesbian-led families, such as infant preference, infertility, and gender and role identifications, correlate with feelings of jealousy between lesbian co-parents.	In-depth interviews	30 lesbian couples (n=60 women)
Pereira DMR, Peçanha LMB, Araújo EC, Sousa AR, Galvão DLS, Lima MFG, Souza MC, Torres KCC. (2024), Brazil [42]	A qualitative, descriptiveand exploratory study.	To analyse the experiences and meanings attributed by trans men to the breastfeeding process in light of the Theory of Social Representations.	Individual interviews with a semistructured script. An objective questionnaire was also applied to characterise the sample.	5 bi/hetero/pan sexual transgender men
Rippey, P. L. F., & Falconi, L. (2017), Canada [33]	A qualitative pilot study of qualitative interviews and surveys.	To explore the relationship between breastfeeding and identity in lesbian-identified families.	Qualitative interviews and a short survey.	4 couples and 2 birth mothers (n=10 participants)
van Amesfoort JE, van Rooij FB, Painter RC, Valkenburg-van den Berg AW, Kreukels BPC, Steensma TD, Huirne JAF, de Groot CJM, Van Mello NM. (2023), The Netherlands [43]	A qualitative study that uses an inductive approach with the constant comparative method.	To explore the needs and barriers of transgender men in family planning, pregnancy, childbirth, puerperium and perinatal care, to improve perinatal care for transgender individuals and their health care providers.	Individual semi-structured interviews.	5 participants who had known to be pregnant and visited the Centre of Expertise on Gender Dysphoria at the Amsterdam University Medical Centers
Ziegler, E., et al. (2026), Canada [44]	A qualitative study followed the tenets of interpretive description.	To explore the experiences of chestfeeding from the perspectives of transgender men and non-binary people, guided by queer phenomenology	Semi-structured interviews were conducted	Eight transgender men and non-binary people, who had recent chestfeeding experiences. (n=8)

**Fig. 2:**
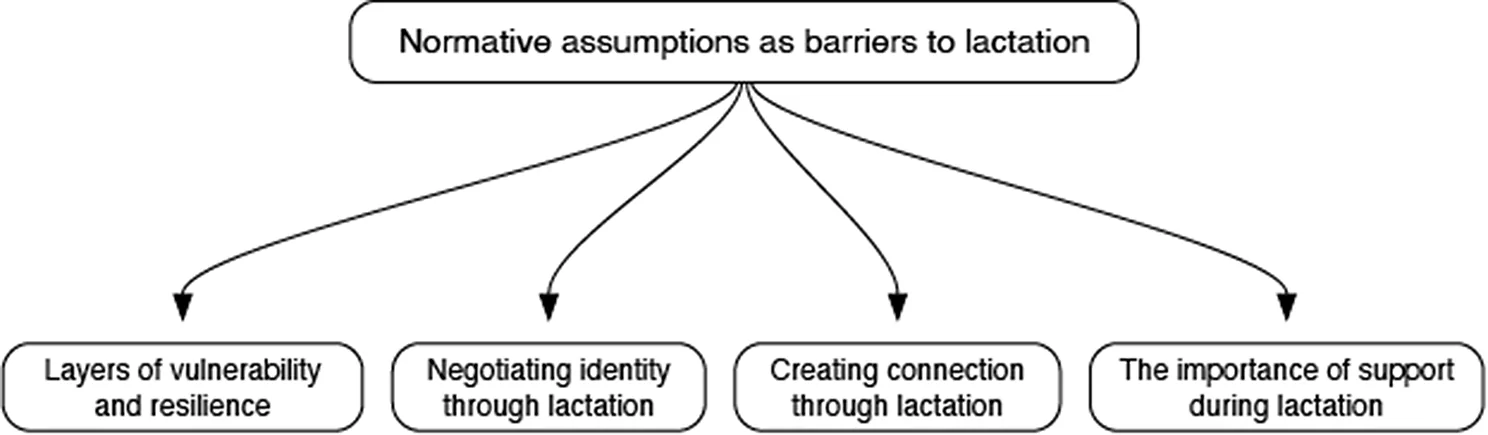
Themes developed from the analysis. SGM parents experience lactation resulting in one core theme, which explains and relates to four subordinate themes

The synthesis of the included studies resulted in one core theme: Normative assumptions as barriers to lactation. The core theme comprised four subordinate themes: Layers of vulnerability and resilience; Negotiating identity through lactation; Creating connection through lactation; and the importance of support during lactation (Fig. 2).

### Normative assumptions as barriers to lactation

The core theme describes how lactation is often perceived by the public as an act tied to specific identity markers, namely, that the lactating parent is (A) heterosexual [34, 35, 40] (B) female [36] and (C) a mother [33, 44]. This aligns with Butler’s queer theoretical critique of dominant societal norms [21], in which heteronormativity and cisnormativity shape assumptions about bodies, gender roles, and parenting. SGM parents may not conform to one or more of these assumptions, which can create barriers to establishing and sustaining lactation and to positive nursing experiences. For some parents, this contributed to a sense of invisibility [33].

Assuming that a parent is heterosexual can render non-gestational parents in same-sex couples, who identify as “mothers” and wish to nurse their children, less visible and less recognised [29, 33, 40]. The second assumption, that the lactating parent is female, fails to acknowledge transmasculine and non-binary parents. In the included studies, transmasculine parents were either treated as female — and therefore assumed able to lactate — or recognised as male and therefore not seen as lactating individuals [30, 36, 44]. The third assumption, that the lactating parent is a mother, overlooks transmasculine and non-binary parents who identify as fathers and still choose to lactate [30, 36, 44]. It also fails to acknowledge the possibility that a child may have more than one lactating mother [33, 40].

### Layers of vulnerability and resilience

Vulnerability was conceptualised as a multidimensional and relational experience. Vulnerability was not simply an individual deficit or state of weakness, but something that was produced or intensified through particular social, relational, and structural circumstances [40].

**Fig. 3:**
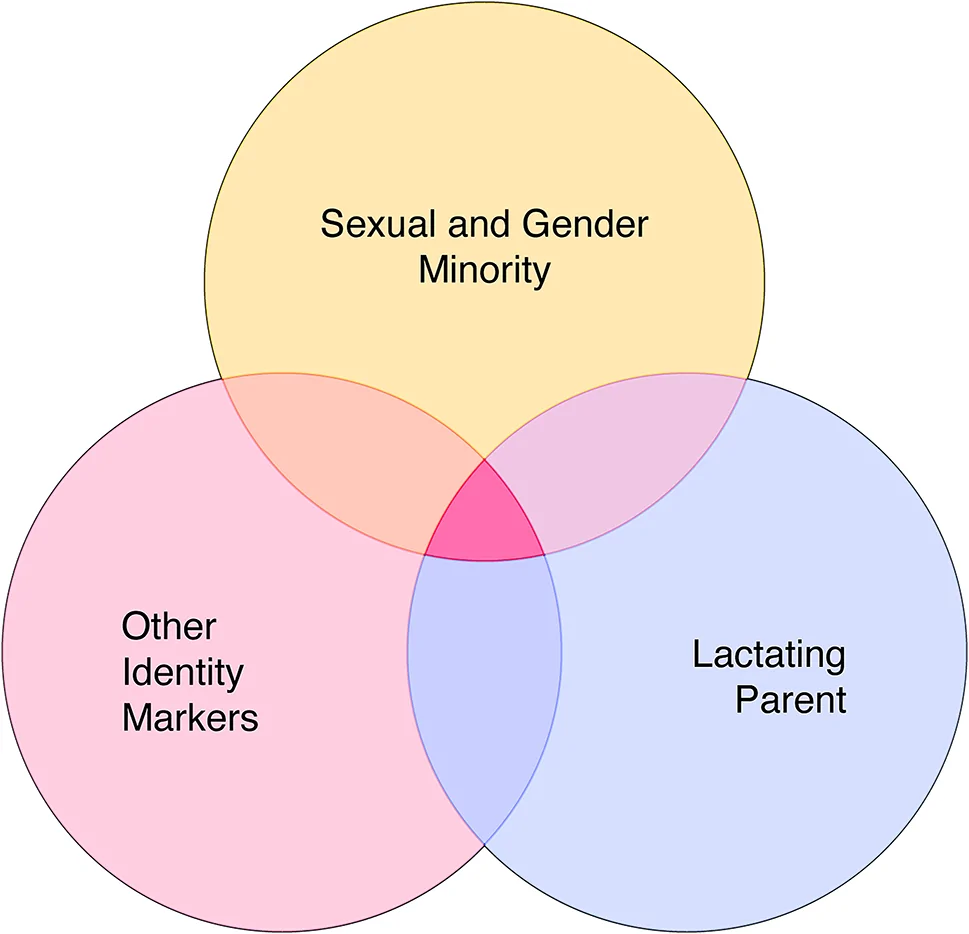
Vulnerability model. The experience of lactation as a SGM parent was influenced by other identity markers. The intersection between being a sexual and gender minority and identifying with another marginalised group, meant that some parents experienced lactation from a more vulnerable place

Lactation can be a vulnerable period because physical, emotional, and practical challenges can reduce confidence and make it harder to establish or continue lactation [36]. For SGM parents, these challenges were often compounded by minority-related stressors, such as anticipating stigma, being misrecognised, or using services that do not reflect diverse bodies and family structures. However, an intersectional perspective emphasises that experiences are shaped by the combined effects of multiple social positions and contexts, rather than by SGM identity or lactation alone [22, 40]. Across the included studies, some SGM parents described entering lactation from a particularly exposed starting point [32, 35–38, 40, 44]. At the same time, vulnerability was not uniform. The findings show substantial variation within SGM populations: many parents described challenges that are common across lactation, but the sources, intensity, and consequences of vulnerability differed, making broad generalisations inappropriate. Based on the synthesis of the included studies, we developed the Vulnerability Model (Fig. 3) to illustrate this complexity.

SGM parents’ lactation experiences were also shaped by other minority identities, such as being Black, Indigenous, and/or a person of colour, which appeared to increase vulnerability further [33, 39]. For Black SGM parents, experiences were influenced by the historical legacy of forced lactation and nursing of enslavers’ children [39]. Other minority positions, such as being Mexican American, could also create barriers to lactation through cultural pressures to use formula [33]. In addition, some vulnerabilities were specific to trans parents [37, 44] and to non-gestational SGM parents (i.e., parents who did not give birth) who wished to lactate [40]. For some, the process of inducing lactation evoked mixed emotions, including frustration, excitement, doubt and hope [28].

In contrast to vulnerability factors linked to SGM lactation experiences, resilience helped parents adapt to challenges. SGM parents who were confident advocates and well informed appeared more likely to have their lactation needs recognised than those with mental health concerns or language barriers, as educational and socioeconomic resources could compensate for healthcare professionals’ limited knowledge, experience and understanding [35, 40]. An affirming and supportive environment also reduced feelings of vulnerability during lactation. Social networks, place of residence, reading habits, and family traditions contributed to resilience by helping parents navigate challenges and cope during difficult periods [39].

### Negotiating identity through lactation

Across the included studies, SGM parents described how their SGM identity shifted over the course of lactation [28, 29, 33, 37, 41, 44]. Parents related to lactation in different ways in relation to their sense of self. For some, being both an SGM parent and lactating prompted a conscious effort to maintain their sexual and/or gender identity; for others, it led to a temporary downplaying or erasure of that identity [33, 37, 39, 42, 43]. Studies published before 2017 suggested that some same-sex parents differentiated parental roles in ways that aligned with more traditional masculine and feminine behaviours [29, 33, 41]. Parents also described tensions when lactation was viewed as central to parenting as an important part of parenting, including having to relinquish the primary nursing role [40, 41] and noticing that children’s preferences could align with the parent perceived as taking a “more nurturing” role [29]. In more recent studies (2019 onward), lactation was also described to disrupt these roles and normative associations, for example through induced lactation, relactation or co-lactation/co-nursing [28, 37]. Transmasculine individuals and their embodied experiences further disrupted normative associations, highlighting how lactating bodies may challenge assumptions about gender and maternal identity [44].

While lactation strengthened parental identity for some [33], SGM identity did not necessarily follow the same trajectory. In the included studies, parents described two contrasting patterns: their SGM identity either became less visible in public settings [33] or was something they deliberately held on to. In the latter case, maintaining SGM identity could also strengthen both SGM identity and the parental role [42]. For lactating and nursing lesbian ciswomen, for example, others often assumed they were heterosexual and had a male partner [33]. Participants also described how normative ideals romanticised and idealised lactation as part of the motherhood, which could exclude father figures and place a disproportionate burden on the mother [36]. Cisnormative expectations made lactation a particularly difficult path for transmasculine parents [36, 44]. Some parents also described internalising dominant assumptions that a lactating parent is heterosexual, female and mother [33]. For some women who identified as female, lactation and nursing were positioned as central to being a “good mother”, providing comfort and care in almost all situations [40]. In one study, lactation was described as more important than being visibly recognised as a sexual minority [33]. For transmasculine and gender-diverse parents, lactation could also conflict with gender expression. Wearing maternity or nursing clothing that felt “hyper-feminine” could cause discomfort [39]. Dressing and lactating in ways that aligned with one’s sexual and/or gender identity often meant departing from conventional nursing practices, which required additional confidence and effort compared with gender-conforming mothers [33].

Studies also reported that some transmasculine parents experienced tensions between their gender identity and ideals of “good parenting”, where lactation was framed as central to attachment parenting [39, 44]. For some, maintaining a masculine gender presentation was as important as the wish to lactate [39]. This could lead to internal conflicts, as their gender identity influenced their decision to initiate and continue lactation. The tension between wanting to provide human milk to their infant and the physiological changes associated with lactation, could intensify gender dysphoria [44]. Transmasculine parents who had not undergone chest masculinisation surgery nevertheless chose to chestfeed their infants [39]. Lactation could also function as a visible marker of sexual and gender minority status [43], as it involved less normative combinations of gender expression; for example, having a beard while lactating. Parents described situations in which lactation-related chest changes affected how others perceived their gender [37]. Some reported discomfort with their chest being observed, as “large” lactating breasts were understood as a cisgender women’s phenomenon [42]. This was especially challenging because lactation can result in prominent chest tissue [37]. For some, binding and using testosterone helped them be perceived as male despite lactating and nursing. Conversely, the absence of stereotypically masculine features (e.g., facial hair) could contribute to lack of recognition of their gender identity [39]. Yet exclusion and negative reactions were described as particularly severe when parents were lactating and “passing” as male [36].

Bodily changes related to lactation did not necessarily trigger gender dysphoria; rather, dysphoria was commonly linked to experiences of being misgendered. Participants noted that pregnancy-related bodily changes were sometimes interpreted as a “male” belly and could be easier to conceal with clothing than lactation-related chest changes [37]. One study described dissociation during lactation, where “shutting off” from the body and “going on autopilot” enabled nursing as a conscious coping strategy [34]. For others, lactation did not diminish their identity as men but was experienced primarily as an act of care for their child [39, 42].

Holding on to one’s identity could also bring positive surprises. This was particularly true for some trans men, who had expected chestfeeding to provoke dysphoria [39, 42]. In one study, parents described their feelings shifting during pregnancy towards a greater sense of calm, and later characterised lactation as physically painful but emotionally rewarding, strengthening the bond with the baby [42]. Some transmasculine parents were surprised to produce milk despite having had chest masculinisation surgery, describing unexpected positive physical and emotional responses even after planning against lactation or taking steps to suppress it [39]. Other accounts suggested that confidence in lactating could increase over time, for example, being unable to nurse a first child due to dysphoria but able to nurse a subsequent child after becoming more comfortable in one’s body [43]. Stopping lactation was described as complex: as lactation ceased, some parents felt they appeared more masculine and were more often perceived as cisgender men and fathers, which could be experienced as both affirmation and erasure of queer identity [43]. Some transmasculine parents also described guilt about not nursing and, at the same time, relief when milk production ended [37].

Cisgender parents described how heteronormative assumptions could create a sense of invisibility during lactation [28, 32]. Some mothers in same-sex relationships reported feeling secure in being openly and “unapologetically” lesbian and initially emphasised that their sexual orientation had little to do with their lactation experiences. They also described strong friendship networks within SGM communities, and for them nursing did not appear to alter their sense of self as lesbians. However, with further reflection, these accounts indicated that sexual orientation did intersect with feeding practices, and that feeding practices could, in turn, shape how sexual identity was experienced [33].

### Creating connection through lactation

Across the studies, SGM parents described multiple motivations for lactation, including meeting the infant’s nutritional needs [39, 42] and enabling emotional closeness between infant and parent [28, 37]. Lactation was often valued for its dual role as both nourishing and nurturing, which could help parents persist despite discomfort and other challenges [42]. However, these motivations alone were not always sufficient to support a sustainable lactation experience. Parents emphasised the importance of emotional and practical support, as lactation could involve significant physical difficulties [36] and was described as “complicated and scary”, particularly when experienced alone [42]. Even in situations of severe pain, such as cracked and bleeding nipples, some parents described nursing as “worth it” [42]. Others expressed fascination with the way human milk adapts to the infant’s needs, describing it as “incredible” [39]. Some parents were unable to lactate but still sought to access to human milk because of its perceived benefits [43].

Parents who pursued induced lactation, relactation, or co-lactation/co-nursing often described closeness with the child as a primary motivation [28, 37]. Induced lactation was framed by both ciswomen and trans women to support a deeper emotional bond with the baby [33, 37]. It was described as an opportunity to participate directly in feeding and to establish an embodied connection with their infant, while simultaneously requiring considerable physical and emotional effort [40]. For transmasculine parents, like lesbian couples and trans women, motivations commonly included bonding and attachment-oriented parenting [39]. In addition, lactation could be experienced as a commitment to caregiving despite others questioning their masculinity [35, 44]. Despite the challenges involved, some parents described the decision to lactate as “simple”, based on perceived health benefits for the infant [39].

Co-lactation/co-nursing was also described to bridge biological differences and strengthen family connection. Although co-nursing could be demanding and time-consuming, it was valued as a shared approach to feeding that enabled parents to distribute responsibility and was associated with positive experiences [32]. It was also described as a means of equalising parenting by enabling shared caregiving and distributing the practical and emotional demands of infant feeding [33]. Parents described co-nursing as deeply rewarding for both the parents and the infant, contributing to emotional, mental, and physical well-being [32]. Overall, for non-gestational parents, the desire to lactate was primarily linked to not having experienced pregnancy, rather than being pursued despite it. For some female partners, lactation offered a way to move towards a primary caregiver identity despite biological constraints [37].

The end of the lactation period was experienced as a significant emotional loss, as it often reduced the felt closeness with the child. Parents who transitioned from co-lactation to lactation by only one parent reported stress and a renewed imbalance in caregiving [28]. Both birth and non-gestational parents described how lactation could shape a child’s preference for one parent over the other. Others attributed the child’s preference to factors beyond lactation, which illustrate how parents drew on different social and biological explanatory models to make sense of parent-child dynamics [29].

Some parents described how not lactating limited their ability to provide the same level of comfort and closeness [33]. For some female partners, not lactating was associated with feelings of exclusion or inadequacy [33, 40], particularly when infant preferences appeared to favour the lactating parent [41]. For non-gestational parents, watching a partner nurse could also be emotionally overwhelming. In studies conducted before induced lactation was available, non-gestational mothers often reported emotional strain linked to being unable to lactate [41]. Jealousy towards the gestational parent’s ability to lactate and nurse were, in part, explained by the lack of role models for lesbian motherhood and limited visibility of alternative parenting arrangements, as some participants described being among the first generation of openly same-sex couples. Some struggled with shifting roles and described having to relinquish earlier expectations of being the one to nurse, which could create identity tensions. Overall, these findings suggest that the embodied connection of lactation can shape early parental identity and bonding, as well as the couple relationship [41].

### The importance of support during lactation

In the included studies, parents often described receiving lactation guidance that was practical in intent but experienced as insensitive. In some accounts, parents were touched on the chest without consent (e.g., “they would just grab and manipulate”), which caused distress during an already challenging period [39]. Lactation experiences were also shaped by the normative contexts in which SGM parents navigated care [28, 32, 33, 35, 36, 40, 44]. These contexts were associated with limited support and inconsistent advice [32, 33, 37, 38, 43], particularly in interactions with healthcare professionals, where institutional routines often did not reflect the diversity of SGM parents’ identities and needs. Such encounters also contributed to experiences of prejudice, including homophobia and transphobia [32, 35, 44]. In response to these normative assumptions, parents described both avoidance and resistance strategies.

Supportive and knowledgeable healthcare professionals could play a crucial role in making nursing more manageable and fostering a more positive lactation experience [37, 39, 42]. Healthcare professionals who demonstrated knowledge of and sensitivity to sexual and gender minority identities were particularly valued, with gender-affirming care identified as an important facilitator of positive lactation experiences. Already during the prenatal period, this was actively sought for [44]. Satisfactory lactation support was also described as contributing to psychological postpartum recovery [38]. However, parents often reported that emotional support from healthcare professionals was inconsistent [37]. One study found that few parents described explicitly positive and affirming encounters in which healthcare professionals encouraged induced lactation [38]. Given low expectations of support, some trans men valued clinicians who openly acknowledged limited knowledge, interpreting this as honesty and a willingness to learn [36]. Parents also described strategies to minimise conflict, including overlooking disrespectful treatment such as the use of incorrect pronouns [35].

Lack of support was the most prominent negative aspect of lactation described by SGM parents in the included studies [38]. Parents reported receiving either practical lactation support without emotional support [37] or emotional support without corresponding practical help [36]. Gaps in either area could have negative consequences. These assumptions were reflected in the use of incorrect pronouns, misgendering, and limited awareness of SGM-specific needs [37, 43, 44]. In several studies, healthcare professionals were described as struggling to respond to SGM parents’ lactation needs, particularly regarding induced lactation for same-sex female couples [32] and lactation among transmasculine or non-binary parents [30, 44].

SGM parents’ limited access to lactation services and particularly emotional support, was associated with phobic attitudes encountered in healthcare settings. Parents described how culturally insensitive care created barriers to appropriate support for transmasculine parents, including dismissive or minimising responses [37, 39, 44]. Healthcare professionals were also reported to ask irrelevant or uncomfortable questions [37, 39]. Transmasculine parents were denied lactation coaching or were laughed at [30]. Such treatment contributed to heightened vulnerability, especially when parents had to expose intimate body parts such as the chest [39] and could lead to avoidance of help-seeking during lactation [30]. Other protective strategies included restricting lactation and nursing to the home environment to reduce exposure to transphobic encounters [42]. Beyond healthcare, transphobia was also described in public settings, including intrusive curiosity, staring, and unauthorised photographing or filming, particularly when trans men nursed while presenting with traditionally masculine features such as facial hair [39]. Some parents also reported fear of violence related to nursing in public [36].

Concealment of trans identity and lactation status could also have harmful consequences. One account described being prescribed medication that was unsafe during nursing, after which the infant became ill [30]. Parents reported that disrespectful and phobic treatment from healthcare professionals could contribute to psychological distress, including gender dysphoria triggered by misgendering [39, 42]. Limited emotional support was also evident when healthcare professionals did not recognise gender dysphoria as a legitimate reason to avoid lactation [35]. For some transmasculine parents, trying to maintain testosterone treatment while nursing was described as emotionally distressing, reflecting broader gaps in recognition and sensitivity within healthcare practice [37, 44]. Parents also described pressure to conform to breastfeeding expectations. In some accounts, healthcare professionals encouraged nursing even when parents were physically unable to lactate [39].

Non-gestational parents who pursued induced lactation or relactation described feeling judged for wanting to breastfeed their infants [28]. Nursing within a same-sex relationship was met with surprise, as others assumed the infant would be nursed by the other mother [32]. Explicit homophobia was also reported, including comments suggesting that lesbians could not practice attachment parenting [33].

Limited practical lactational support was linked to healthcare professionals’ difficulty locating clear, evidence-based guidance. Some healthcare professionals were described as demonstrating a willingness to learn by acknowledging uncertainty, discussing it openly with parents, and collaboratively weighing risks and benefits [30, 43]. However, parents emphasised that acceptance alone was not the same as competence in meeting SGM-specific care needs [32]. Many healthcare professionals were described as unaware that non-gestational parents can lactate, and parents highlighted the need for anticipatory guidance, including what to expect, when to seek help, and when to consider stopping [32]. Parents also reported inconsistent or outdated advice, as well as limited knowledge about co-nursing and induced lactation [37, 38]. In some studies, non-gestational parents described uncertainty about the advice they received [38], despite needing specialised support, for example, guidance on dysphoric milk ejection reflex or on the substantial off-label medication sometimes required to maintain lactation for trans women [37]. Overall, the included studies point to gaps in cultural competence, knowledge, protocols and resources needed to provide inclusive lactation support for SGM parents [30, 32, 35, 39]. Limited practical support, experiences of exclusion were also described as contributing to physical health concerns during lactation [35, 38].

Healthcare professionals were described as unprepared when transmasculine parents who had undergone chest masculinisation surgery began lactating [34]. Healthcare professionals did not anticipate lactogenesis after chest masculinisation surgery and were reported to provide little or no lactation information [35, 37, 39], partly because they were unsure how such surgery might affect nursing capacity [37]. Transmasculine parents also described that surgeons rarely initiated conversations about future infant feeding when planning or performing chest masculinisation surgery [39]. Due to the limited knowledge and practical support, parents reported a range of negative physical and informational experiences, including chest tissue regrowth during pregnancy [39, 43], undiagnosed mastitis [35, 39], lack of information about donor milk options [39], and uncertainty about the safety of lactation while using testosterone [30, 36, 37, 44].

The ability to pursue induced lactation or co-nursing was often described as dependent on parents’ compensatory resources [32, 40]. Anticipating that lactation would be difficult, parents reported seeking information to educate themselves by buying books and contacting non-profit breastfeeding organisations [32]. The internet and digital influencers of parenting were, for some SGM parents, identified as the main source of information [40, 44], possibly explained because of absent health professionals [40]. Transmasculine parents turned to peers for medical advice, raising concerns about safety when community guidance differed from advice provided in healthcare settings [35–37]. Parents who had not undergone chest masculinisation surgery also described taking proactive steps during pregnancy to prepare for lactation and relying heavily on personal networks for emotional support, given limited guidance and medical information from healthcare professionals [34, 39].

## Discussion

This meta-ethnography shows that SGM parents often had to navigate intersecting forms of normativity, prejudice, and structural exclusion during lactation. Limited knowledge and support among healthcare professionals may also reflect broader societal norms and institutional practices. Vulnerability was particularly evident in highly gendered maternity care settings, including labour, birth, and postpartum care, where policies, language, routine practices and assumptions, and limited professional training could marginalise SGM parents.

The findings suggest that SGM parents’ lactation experiences are shaped by both internal and external stressors, including fear of misgendering, anticipated rejection, and prior encounters with discrimination. These accounts align with the concept of minority stress, which describes the cumulative psychological burden of living in a stigmatising environment [45]. The qualitative evidence also indicates that recurring microaggressions, often subtle, can contribute to exclusion and undermine confidence in early parenthood. Subtle forms of discrimination may also be harmful, particularly when repeated or encountered in settings where support is expected [46]. These findings highlight the need for inclusive lactation support that actively mitigates the effects of minority stress.

Because gender and sexuality are understood as socially constructed, the norms that shape SGM parents’ lactation experiences can also be reshaped and contested [47]. This dynamic is evident in our synthesis: differences between more recent studies and earlier work suggest that SGM parenting roles, and their relationship to lactation, are not fixed but adaptable over time. This aligns with queer theory [21], which conceptualises norms around parenting, gender, and lactation as negotiable rather than static, and highlights how less normative ways of parenting and lactating can disrupt and reconfigure dominant expectations.

As SGM parents move beyond inherited heteronormative perspectives, their lactation experiences can be understood as “queering” the lactating body and expanding what counts as a lactating parent beyond normative constraints. Lactation can contribute to disrupting traditional expectations through less normative uses of the body. Accounts from transmasculine parents who chose to nurse illustrate that bodily function does not determine gender. These experiences may also broaden cultural understandings of fatherhood by showing how fathers can engage in caring, embodied forms of parenting that challenge heteronormative norms.

Lactation intersects with being a sexual and gender minority. Although human milk provision can be understood as work, lactating bodies are often devalued within patriarchal systems. Postpartum bodies are expected to lactate, yet they are not consistently supported, prioritised, or valued [48]. At the same time, when a body is read as male, lactation may be delegitimised or rendered invisible. Femmephobia refers to the devaluation and regulation of femininity, and, when combined with transphobia, can contribute to the erasure of transmasculine people [49]. Sexual orientation adds another layer to these dynamics. Lactation may occur independently or with partner support, but dominant assumptions typically position the partner as male and heterosexual. These assumptions contribute to the lack of a defined and socially recognised role for the non-gestational parent [50], which becomes particularly consequential when that parent needs lactation-related support and care.

These overlapping forms of exclusion can compound invisibility and structural marginalisation in healthcare, social policy, and dominant narratives of parenthood. Accordingly, SGM parents’ vulnerability in relation to lactation needs to be understood through an intersectional perspective, which attends to how multiple dimensions of identity interact and reinforce one another [22].

Queer theory critiques normative systems and create space for identities that do not fit traditional categories [21]. Because SGM parents’ lactation practices challenge dominant expectations, they can be perceived as disrupting the status quo. For some parents, lactation therefore became an act of resistance [51] to normative assumptions about gender and parenting. In these accounts, lactation was both a biological function and a practice through which parenting roles and family relations could be reconfigured. As normative boundaries shift, lactation can take on political meaning by prompting reflection on power dynamics within the family. This study also suggests that such acts of resistance can contribute to cultural change and highlight the contingency of what is often framed as “natural”. In some accounts, a satisfying lactation experience supported identity formation during the transition to parenthood, as becoming a parent was described as actively shaped through embodied and relational experiences around childbirth.

### Strengths and limitations

Using queer theoretical and intersectional perspectives provided an analytical perspective which strengthened the transparency and credibility of the interpretation and synthesis. Selecting databases that reflect the interdisciplinary nature of the research question is also important [46]. PubMed, Cinahl and Scopus were chosen to capture multidisciplinary perspectives, and the broad search strategy increased the likelihood of identifying relevant qualitative studies [47]. Comparing studies published before 2017 with those from 2019 onward helped situate the findings over time and supported interpretation in relation to changing norms.

Overly narrow eligibility criteria can exclude important voices [48]. Limiting inclusion to English-language studies also risks under-representing non-English and non-Western perspectives, which is particularly relevant given that SGM experiences are culturally situated. The heterogeneity of analytical approaches across the included studies is another limitation, as differences in analytical frameworks and levels of interpretation may have influenced the concepts available for translation and synthesis. Three included studies did not report ethics committee approval [28–30]; we nevertheless included them because these contributed to understanding the phenomenon. In meta-ethnography, study value is commonly assessed by its contribution to conceptual understandings and interpretation rather than by methodological quality alone [19].

Queer theory and intersectional perspectives do not provide a linear route from theory to practice, which may limit the direct translation of findings into clinical guidance and introduce interpretive complexity. However, this limitation is not unique to these perspectives. Other commonly used frameworks in nursing and midwifery, such as humanistic care and midwifery care theories, also rest on abstract principles that can be operationalised in different ways and lack consistently applied, practical definitions or clearly specified epistemological foundations [49] This highlights an ongoing need for theoretical development and for work that translates conceptual understandings into actionable models for maternity care [50].

### Practical implications

Equity is a recurring and complex theme in the literature, as social norms can contribute to the exclusion of marginalised groups within health systems [51]. This meta-ethnography suggests that SGM parents may experience differences in treatment and care, linked to navigating healthcare environments shaped by heteronormativity and cisnormativity. Normative assumptions about gender, sexuality and parenting can affect the whole family. Future research should examine how these inequities arise and identify actions at clinical, organisational, and policy levels that can promote equitable and inclusive care.

With cultural competence, healthcare professionals may better recognise how SGM parents’ lactation experiences can diverge from hetero- and cisnormative expectations. Framing SGM parents as having a distinct “minority culture” risks reinforcing stereotypes. Culturally informed care should instead centre the individual’s lived experience and priorities [52], to enable accurate, person-centred support throughout the lactation period. Inclusive lactation care requires changes in clinical practices, education and communication. Based on our findings, it is recommended to include targeted training and clear guidance to strengthen healthcare professionals’ capacity to provide affirming, individualised lactation support across diverse gender identities and sexual orientations.

## Conclusion

This meta-ethnography shows that SGM parents’ lactation experiences are shaped by hetero- and cisnormative assumptions that position the lactating parent as heterosexual, female, and a mother. When parents’ identities and family structures do not fit these expectations, they can encounter misrecognition, inconsistent information, and inadequate support, which may undermine a positive lactation experience. An intersectional perspective further highlights how these challenges can be compounded by other dimensions of identity and social position. Supporting SGM parents to lactate on their own terms is both a health and equity issue. The findings point to structural gaps in maternity and lactation care and emphasise the need for clear guidelines, targeted training, and affirming, person-centred healthcare services. Future research should examine longer-term outcomes, including mental health and parent–infant attachment, and evaluate strategies that improve the quality and equity of lactation support for SGM parents.

## Supplementary Information

Below is the link to the electronic supplementary material.

Supplementary Material 1

## Data Availability

No datasets were generated or analysed during the current study.

## Abbreviations

CASP: Critical appraisal skills programme
PEO: Population, exposure, outcome
SGM: Sexual and gender minority
