# Supplementary material for: Sexual and gender minority parents’ experiences of lactation: a meta-ethnographic review
Source: Int Breastfeed J. 2026 Sep 21;21:88. doi: 10.1186/s13006-026-00897-8 (PMC13595532; doi:10.1186/s13006-026-00897-8)
Supplement: Supplementary file 1 [file 13006_2026_897_MOESM1_ESM.pdf]

Supplementary file 1
The eMERGe meta-ethnography reporting guidance
From: Improving reporting of meta-ethnography: the eMERGe reporting guidance

No.	Criteria Headings	Reporting Criteria
Phase 1—Selecting meta-ethnography and getting started		
Introduction		
1	Rationale and context for the meta-ethnography	*Describe the gap in research or knowledge to be filled by the meta-ethnography, and the wider context of the meta-ethnography*<br>The rationale stems from the gap in existing research regarding parenting and lactation support for sexual and gender minority (SGM) individuals. Filling this gap is crucial for facilitating a more equitable and inclusive healthcare system, and for deepening our understanding of the unique challenges SGM parents face.<br>This work offers valuable insights into how current support structures can be improved, and highlights the role of societal and institutional norms in shaping the parental experiences of SGM individuals.
2	Aim(s) of the meta-ethnography	*Describe the meta-ethnography aim(s)*<br>The aim of this study is to synthesise and interpret existing qualitative research on SGM parents' experiences of lactation.
3	Focus of the meta-ethnography	*Describe the meta-ethnography review question(s) (or objectives)*<br>The questions addresses the intersection of experiences of lactation, parenting and SGM issues that are often overlooked in research.<br>• How do SGM parents perceive the support they receive in connection with lactation?<br>• How does the perceived support affect lactation and parenting?
4	Rationale for using meta-ethnography	*Explain why meta-ethnography was considered the most appropriate qualitative synthesis methodology*<br>Meta-ethnography enables the development of higher-order interpretations, making it particularly well-suited to exploring complex, nuanced, and socially embedded

		experiences, such as those of sexual and gender minority (SGM) parents navigating lactation support.<br>It offers a deeper understanding of how cultural, institutional, and interpersonal factors shape experiences of care and identity.
Phase 2—Deciding what is relevant		
Methods		
5	Search strategy	*Describe the rationale for the literature search strategy*<br>The literature search strategy was designed using the PEO (Population; Exposure; Outcome) framework to ensure a structured and targeted approach. This helped define the search terms and inclusion criteria in a way that aligned with the research focus on sexual and gender minority (SGM) parents and their lactation experiences.<br>A broad definition of both lactation and SGM identities was deliberately chosen to capture a wide range of relevant studies and perspectives, given the limited research available in this area. No publication date restrictions were applied to maximize the scope and allow for exploration of how societal and gender norms may have influenced SGM lactation experiences over time.<br>The search strategy was developed in collaboration with librarians at the University of Gothenburg to ensure comprehensiveness and relevance. The selected databases—PubMed, CINAHL, and Scopus—were chosen for their coverage of medical, nursing, and interdisciplinary health research. Both manual and automatic methods were used to remove duplicates and maintain the integrity of the dataset.
6	Search processes	*Describe how the literature searching was carried out and by whom*<br>The literature search was carried out by MCB in collaboration with librarians from the Gothenburg University Library. Search terms were developed using the PEO framework and tailored for each database. Searches were conducted in PubMed, CINAHL, and Scopus between February 1–10, 2025. The search strings included broad terms related to both lactation and sexual and gender minority identities. Duplicates were removed both manually and using database tools.

7	Selecting primary studies	*Describe the process of study screening and selection, and who was involved*<br>The screening and selection process involved MCB as reviewer who independently assessed all 273 abstracts against predefined inclusion criteria. No exclusion criteria were applied. Any uncertainties were resolved through discussion with the study supervisor. Of the screened abstracts, 28 full-text articles were reviewed, and 14 studies met the criteria for inclusion in the final synthesis. The initial search was conducted in December 2024, with an updated search in February 2025.
Findings		
8	Outcome of study selection	*Describe the results of study searches and screening*<br>The initial and updated database searches yielded a total of 273 articles after duplicates were removed. No additional studies were identified through other search methods. All abstracts were screened by MCB using predefined inclusion criteria. Of these, 28 full-text articles were assessed for eligibility. Following full-text review, 14 studies met the inclusion criteria and were included in the final meta-ethnography. The screening process is detailed in a PRISMA flowchart.
Phase 3—Reading included studies		
Methods		
9	Reading and data extraction approach	*Describe the reading and data extraction method and processes*<br>Critical Reading and Data Extraction: Key data from each study were extracted using the CASP (Critical Appraisal Skills Programme) predesigned template.<br>The selected studies were read repeatedly and thoroughly. This phase involved identifying key themes, metaphors, and concepts, with careful attention to how support experiences were shaped by intersections of gender, sexuality, race, and other identity markers.
Findings		

10	Presenting characteristics of included studies	*Describe characteristics of the included studies*<br>A total of 14 articles were included in the final review (Table 4). The studies were conducted in Brazil (n=2), Canada (n=2), Spain (n=1), Sweden (n=3), The Netherlands (n=1), United Kingdom (n=1) and in the USA (n=4). Overall, these studies represent the views of 242 SGM parents, a group that consists of female same-sex couples, as well as transmasculine, transfeminine and nonbinary individuals.<br>The included studies were conducted between 2008 and 2024. Data was primarily collected through qualitative interviews, including in-depth, semi-structured, and reflective formats. In some cases, written responses from open-ended survey questions were also analysed.<br>The studies used a variety of analytical approaches, including both inductive and deductive methods, and were grounded in traditions such as thematic analysis, grounded theory, ethnography, and interpretive description. One study applied mixed methods design, and another was conducted as a pilot study.
Phase 4—Determining how studies are related		
Methods		
11	Process for determining how studies are related	*Describe the methods and processes for determining how the included studies are related:*<br>*- Which aspects of studies were compared AND How the studies were compared*<br>Each study was evaluated for methodological quality to determine its reliability and relevance in the synthesis. This step helped weigh the strength of each study's contribution.<br>Key concepts across studies were compared side by side. Recurring patterns and differences in how support for SGM parents was experienced were identified and grouped.<br>An intersectional and queer theoretical lens was applied to interpret how the themes intersected. This allowed the synthesis to consider variations in experience based on race, gender identity, sexuality, and social power structures.<br>A secondary reviewer (the study supervisor) reviewed one of the articles to enhance reliability and consistency in the comparison process.
Findings		

12	Outcome of relating studies	*Describe how studies relate to each other*<br>The included studies relate to each other through their shared exploration of how sexual and gender minority (SGM) parents experience lactation.<br>In synthesis, the studies complement one another by offering different yet overlapping perspectives on how normative barriers impact lactation. While each study emphasized different facets, they collectively contribute to a coherent understanding of the lactation experiences among SGM parents.
Phase 5—Translating studies into one another		
Methods		
13	Process of translating studies	*Describe the methods of translation:*<br>*- Describe steps taken to preserve the context and meaning of the relationships between concepts within and across studies- Describe how the reciprocal and refutational translations were conducted- Describe how potential alternative interpretations or explanations were considered in the translations*<br>The translation process followed phase five of Noblit and Hare's (1988) meta-ethnographic method: Translating the studies into one another. This phase involved comparing key concepts and themes across the included studies. The process was interpretative, and deeply rooted in intersectional and queer theoretical frameworks, which helped highlight the complexity and power structures embedded in the findings.
Findings		
14	Outcome of translation	*Describe the interpretive findings of the translation.*<br>The interpretive findings of the translation led to the development of the following key themes: Identity Markers, Bonding, Gender Roles, Lack of Knowledge, Lack of Provider Support, Prejudice, Compensatory Resources, and Sacrifice and Inner Conflict. Each theme represents a distinct aspect of SGM parents' experiences with lactation.
Phase 6—Synthesizing translations		
Methods		

15	Synthesis process	*Describe the methods used to develop overarching concepts ("synthesised translations")Describe how potential alternative interpretations or explanations were considered in the synthesis.*<br>Interpretations were discussed with the secondary reviewer to challenge assumptions, refine thematic boundaries, and ensure a nuanced, critical understanding of the data. Alternative interpretations were considered by engaging with contradictions and differing findings across studies. Instead of aiming for consensus, the analysis embraced complexity. For example, some SGM parents found lactation affirming, while others experienced it as distressing. These differences were seen as reflections of broader social and structural factors shaping queer parenting and lactation.
Findings		
16	Outcome of synthesis process	*Describe the new theory, conceptual framework, model, configuration, or interpretation of data developed from the synthesis.*<br>Through the process of meta-ethnographic synthesis, one core theme emerged across the studies: Normative assumptions as barriers to lactation.
Phase 7—Expressing the synthesis		
Discussion		
17	Summary of findings	*Summarize the main interpretive findings of the translation and synthesis and compare them to existing literature*<br>The synthesis of the included studies resulted in one core theme: Normative assumptions as barriers to lactation. The core theme explains and relates to four subordinate themes: Layers of vulnerability, Negotiating identity through lactation, Creating connection through lactation and The importance of support during lactation.
18	Strengths, limitations, and reflexivity	*Reflect on and describe the strengths and limitations of the synthesis:*<br>*- Methodological aspects—for example, describe how the synthesis findings were influenced by the nature of the included studies and how the meta-ethnography was conducted.- Reflexivity—for example, the impact of the research team on the synthesis findings*

		A subjective assessment and interpretation of the result are, to some extent, always present. The identity and perspective of MCB inevitably influenced interpretation. The queer theoretical and intersectional perspectives may therefore strengthen validity and credibility in interpreting and synthesizing the result. The theoretical frameworks of choice are particularly important from an ethical standpoint, as they aid to avoid generalizations and reassure that the diversity of SGM parents' experiences and perspectives are well considered.
19	Recommendations and conclusions	*Describe the implications of the synthesis*<br>The synthesis highlights how normative assumptions around gender, parenthood, and lactation can act as barriers for SGM parents. These assumptions shape both healthcare encounters and social support, often resulting in exclusion, misunderstanding, or inadequate care. By identifying these patterns, the findings point to a need for more inclusive, affirming healthcare practices and support systems that recognise the diverse experiences of SGM parents. For future research, the synthesis reveals gaps in knowledge, particularly around trans and non-binary parents, and calls for more intersectional studies that consider race, class, and other structural factors alongside gender and sexuality.

Supplementary file 2.

Databases, search strings and search results

Database<br>Date	Search string	Search results
PubMed<br>20.08.2026	(Sexual and Gender Minorit* OR Homosexual* OR Gender identity OR Transgender Person* OR Same-sex Parent* OR Same-sex mother* OR Same-sex father* OR queer OR transgender OR non-binary OR lesbian OR lgbtq OR lgbtqia OR Gender Minorit* OR Sexual Minorit*) AND (breast feeding OR lactation OR co-lactation OR lactation, induced OR Galactogogues OR Domperidone)	217
Cinahl<br>20.08.2026	(Sexual and Gender Minorit* OR Homosexual* OR Gender identity OR Transgender Person* OR Same-sex Parent* OR Same-sex mother* OR Same-sex father* OR queer OR transgender OR non-binary OR lesbian OR lgbtq OR lgbtqia OR Gender Minorit* OR Sexual Minorit*) AND (breast feeding OR lactation OR co-lactation OR lactation, induced OR Galactogogues OR Domperidone)	130
Scopus<br>20.08.2026	"sexual and gender minorit*" OR homosexual* OR "gender identity" OR "transgender person*" OR "same-sex parent*" OR “Same-sex mother*” OR “Same-sex father*” OR queer OR transgender OR non-binary OR lesbian OR Igbtq OR Igbtqia OR “Gender Minorit*” OR "sexual minorit*"<br><br>AND<br><br>"Breast feeding" OR breastfeeding OR lactation OR co-lactation OR "lactation, and induced" OR galactogogues OR domperidone OR "induced lactation" OR "milk ejection" OR co-nursing	351

Supplementary file 3.
Excluded articles and reasons

**Wrong population**<br>Does not address SGM parents	Bozlak, C. T., Ruland, L., Eskew, B., Spence, M., & Dennison, B. A. (2024). Breastfeeding disparities and recommended strategies to end them in New York. Preventive Medicine Reports, 47, 102881. https://doi.org/10.1016/j.pmedr.2024.102881
**Irrelevant experience**<br>No lactational experience	Cohen, N., & Oreg, A. (2025). "I am a guest man in a world of women": The lived experiences of gay fathers utilizing human milk donations for their babies. *Social Science & Medicine*, *365*, 117567. https://doi.org/10.1016/j.socscimed.2024.117567
**Study design**<br>Case report / quantitative aspects	Delgado D, Stellwagen L, McCune S, Sejane K, Bode L. Experience of Induced Lactation in a Transgender Woman: Analysis of Human Milk and a Suggested Protocol. Breastfeed Med. 2023 Nov;18(11):888-893. doi: 10.1089/bfm.2023.0197. Epub 2023 Nov 1. PMID: 37910800.
**Study design**<br>Case report	Greenfield, M. (2022). Lack of policy consideration for breastfeeding co-mothers in maternity services. British Journal of Midwifery, 30(9), 526-530. https://doi.org/10.12968/bjom.2022.30.9.526
**Study design**<br>Case report	Ikebukuro S, Tanaka M, Kaneko M, Date M, Tanaka S, Wakabayashi H, Murase M, Ninomiya N, Kamiya T, Ogawa M, Shiojiri D, Shirato N, Sekiguchi Y, Sekizawa A, Nakatsuka M, Gatanaga H, Mizuno K. Induced lactation in a transgender woman: case report. Int Breastfeed J. 2024 Sep 19;19(1):66. doi: 10.1186/s13006-024-00675-4. PMID: 39300546; PMCID: PMC11411746.

Supplementary file 4.
The number of articles included in the final review: 16
Assessment structured according to the CASP Qualitative Studies Checklist.

Quality Assessment

**Article**	**Section A - Are the results valid?**	**Section B: What are the results?**	**Section C: Will the results help locally?**	**APPRAISAL SUMMARY:**
	1. Was there a clear statement of the aims of the research?<br>2. Is a qualitative methodology appropriate?<br>3. Was the research design appropriate to address the aims of the research?<br>4. Was the recruitment strategy appropriate to the aims of the research?<br>5. Was the data collected in a way that addressed the research issue?<br>6. Has the relationship between researcher	7. Have ethical issues been taken into consideration?<br>8. Was the data analysis sufficiently rigorous?<br>9. Is there a clear statement of findings?	10. How valuable is the research?	Positive/Methodologically sound<br>Negative/Relatively poor methodology<br>Unknowns

	and participants been adequately considered?			
**1. Cazorla-Ortiz G, Galbany-Estragués P, Obregón-Gutiérrez N, Goberna-Tricas J.**<br><br>**Understanding the Challenges of Induction of Lactation and Relactation for Non-Gestating Spanish Mothers.**<br><br>Spain<br>Nine women (three same sex couples) (n=9)	1. Yes. <u>Aim</u>: To describe and interpret the challenges faced by mothers who undergo induced breastfeeding and relactation for adopted infants, infants born via surrogacy, and infants born to same-sex female partners.<br><br>2. Yes<br><br>3.Yes<br><br>4.Yes. <u>Recruitment</u>: Author 1 contacted breastfeeding support groups and lactation support providers to identify the first participant. The remaining participants were identified with snowball sampling. Ten women were identified, and nine of these met the inclusion criteria. The nine women were contacted via email to offer study details, provide a consent form guaranteeing data confidentiality, and to commit to informing participants of the study results. The sample was	7.Unknown. <u>Ethical consideration</u>: Participants' names were substituted with the letter "P" (participant), followed by a number. Author 1 saved the transcribed interviews on a personal hard drive to maintain participants' confidentiality. Transcripts were to be destroyed after publication of the study.<br><br>8. Yes.<u>Data analysis</u>: Semi-structured interviews were conducted using open ended questions that addressed difficulties during the process, motivational and demotivational factors, feelings and thoughts, and an evaluation of the entire process. The interview guide was organized around the three phases of induction and relactation, the interview questions followed classic studies in which researchers have laid the groundwork for understanding the	10.Yes. <u>Valuability</u>: At a time when people use a range of modes of reproduction to create their families, health professionals need to know about induced lactation and relactation in order to provide proper guidance and assistance. Researchers and practitioners can use our study to better understand the lived experiences of women during induced lactation and relactation, and health professionals and lactation support providers can draw on it when they offer care during these processes.<br><br><u>Limitations</u>: Overall, the small size of the sample limits the researchers' ability to make definitive claims or generalize the results to other settings. The sample may appear to be skewed toward women with a high educational level. However, this characteristic is probably	Unknowns<br><br>*No approval has been sought from the ethics committee.*

	small due to the small population size and the difficulty of recruitment. Recruiting participants ceased when reaching data saturation.<br><br>5.Yes. Data collection: All participants gave their consent before being interviewed.<br>All data were collected between October 2014 and May<br>2017. Semi-structured interviews were conducted using open-ended questions that addressed difficulties during the process, motivational and demotivational factors, feelings and thoughts, and an evaluation of the entire process.<br><br>6. Yes.	experiences of women undergoing these processes.<br><br>9. Yes.Findings:<br>• The main motivation for undergoing induced lactation or relactation was to strengthen closeness between the mother and child through breastfeeding. Participants reported that breastfeeding cessation diminished this feeling of closeness.<br>• During the processes of induced lactation and relactation, the key challenges for women were physical difficulties, lack of information, lack of support from professionals, and doubts about whether they would have a successful outcome and be able to breastfeed their babies.<br>• Women who underwent induced lactation and were therefore breastfeeding for the first time experienced problems with their breasts. Women undergoing relactation did not have problems with their breasts.	general to women who choose induced relactation or<br>relactation, and therefore not a sampling error. The use of snowball sampling may have skewed the sample toward women who were similar to each other. Finally, the authors' own positioning as nurses and nurse–midwives—<br>and therefore recognizable as people in favor of breastfeeding—could have affected the participants' responses and the researchers' interpretations.	

**2. Falck FAOK, Dhejne CMU, Frisén LMM, Armuand GM.**<br><br>**Subjective Experiences of Pregnancy, Delivery, and Nursing in Transgender Men and Non-Binary Individuals: A Qualitative Analysis of Gender and Mental Health Concerns. Arch Sex Behav.**<br><br>Sweden<br>Twelve transgender men/non-binary individuals (n=12)	1. Yes. Aim:To examine how gestational gender-diverse individuals in Sweden experience the process of planning and undergoing pregnancy, delivery, and nursing.<br><br>2. Yes<br><br>3.Yes<br><br>4.Yes. Recruitment: Participants were recruited through purposive sampling. The community organizations RFSL and RFSL Youth helped recruit participants to the study. Potential participants were then contacted over email with written information about the study and its purpose. This information was also provided in written form as well as orally. Potential participants were also informed about what procedures were in place to ensure confidentiality. After they had been able to ask questions, they confirmed their consent to participate in writing.	7.Yes. Ethical consideration: The study was conducted according to the 1964 Helsinki Declaration and its later amendments (World Medical Association, 2013). Approval by the Regional Ethical Review Board of Stockholm, Sweden, was obtained before study initiation (Archive number. 2016/231–31/5).<br><br>8. Yes.Data analysis: The data was analyzed using thematic content analysis using NVivo software. Qualitative research recommendations and established criteria for the analysis and presentation of unstructured interviews were used. After transcription, all texts were read three times for a preliminary overview. Phrases relevant for the study aim were then identified, coded, and categorized based on similar content, creating main and sub-categories. The transcripts were reread 4–6 additional times, going back and forth between the interview transcripts and the	10.Yes. Valuability: Whereas some gestational transgender men and non-binary individuals find pregnancy, childbirth and nursing fulfilling and in line with their gender identity, others suffer substantial gender dysphoria and mental health problems due to physical changes during pregnancy, the cessation of testosterone, as well as gender stereotypes that associate pregnancy with femininity. Some of these health adversities may be prevented by initiatives to normalize pregnancy in gender-diverse individuals, as well as efforts to strengthen gender congruence prior to or during pregnancy. Such attempts may include the use of role models as well as access to gender-affirming treatment and information on what pregnancy as transgender may be like. These results call for a timely treatment of gender dysphoria as well as comprehensive reproductive healthcare services that are adapted to the needs and	Positive/Methodologically sound

	5.Yes. Data collection: Twelve in-depth face-to-face interviews were carried out from December 2016 to January 2019. Interviews lasted 55–112 min (mean, 83 min), were digitally recorded, and transcribed verbatim. They were conducted two months to two years after the last childbirth of each of the 12 participants. The location of the interviews was chosen by the participants, and the primary author traveled across Sweden to conduct them, reaching urban and rural areas.**Interactions with healthcare providers have been presented in a separate article** (Falck et al., 2021) and are therefore not the focus of this text.<br><br>6. Yes.	categories to avoid misinterpretations and loss of information, deepen, merge, and regroup the categories, and develop and overarching theme based on the data. The first author performed all interviews, the initial coding of the data, and took the lead in the analysis. Once preliminary codes and categories had been formulated, the final author took part in the analysis to help refine and regroup these. The second and third authors were then invited to discuss the data to reach final consensus on main and subcategories as well as the overarching theme.<br><br>9. Yes.Findings:The analysis resulted in one overarching theme: sustaining gender congruence during pregnancy and three main categories:<br>(1) considering pregnancy<br>(2) undergoing pregnancy and childbirth<br>(3) postnatal reflections.	realities of gender-diverse individuals. Laws and policies that limit stigma, discrimination and violence toward gender-diverse individuals and allow them a variety of ways to reach parenthood are also warranted. Furthermore, the risk and management of perinatal mental health problems among gestational transgender men and non-binary individuals require further attention in research and clinical practice.<br><br>Limitations: An important limitation is that the study only included individuals who had carried a pregnancy to term, excluding those who did not have any children due to abortion or miscarriage. This may have resulted in an overly optimistic picture of what pregnancy as gender diverse may be like. Furthermore, none of the participants had planned their pregnancy with a cis-gendered female partner. This limits the analysis of how gender norms affect the decision to	

			undergo pregnancy. However, the fact that we could not identify any gender-diverse individual who had undergone pregnancy while having a female partner may also be an indication of the norm that women bear children. When we recruited participants to this study, a couple of individuals approached us to say that although they wanted to participate in the study, they could not do so due to traumatic memories of giving birth. It is likely that their experiences would have added additional information concerning mental health challenges, the occurrence and management of obstetric complications, as well as gender norms in obstetric care. **A more detailed account of how participants experienced and handled the interactions with healthcare providers, including of how distrust of healthcare providers may extend to maternal and obstetrical care, has been discussed in a**	

			**separate article** (Falck et al., 2021) and is therefore not in focus here.	
3. **Falck, F., Frisén, L., Dhejne, C., & Armuand, G.**<br><br>**Undergoing pregnancy and childbirth as trans masculine in Sweden: Experiencing and dealing with structural discrimination, gender norms, and microaggressions in antenatal care, delivery, and gender clinics.**<br><br>Sweden<br>same individuals as in previous study	1.Yes. <u>Aim</u>: To investigate how trans masculine individuals experience healthcare encounters in connection with pregnancy, delivery and nursing, in a setting where mandatory sterilization to change legal gender was recently removed.<br><br>2. Yes<br><br>3.Yes<br><br>4.Yes. <u>Recruitment</u>:The organizations RFSL and RFSL Youth, transgender experts, gender clinics and former study participants recruited individuals to the study.<br><br>5.Yes..<u>Data collection</u>: Twelve in-depth face-to-face interviews were carried out across Sweden from December 2016 to January 2019. The first author, a resident in psychiatry with several years of clinical	7.Yes. <u>Ethical consideration</u>: The Regional Ethical Review Board of Stockholm, Sweden approved the study, which was performed according to the 1964 Helsinki declaration and its later amendments.<br><br>Before inclusion oral and written study information was provided and participants signed a consent form. The interviewer was not involved in the healthcare of any participant at the time of the interviews or thereafter. Ethical risks were discussed with RFSL and RFSL Youth. Since interviews could invoke traumatic memories, referral procedures for psychological counseling were established. However, no participant needed these services.<br><br>8. Yes.<u>Data analysis</u>:Data was analyzed using inductive thematic content analysis. Inductive analysis was selected, as knowledge on	10.Yes. <u>Valuability</u>:The findings call for individualized, inclusive, respectful and knowledgeable healthcare that does not treat a masculine identity and pregnancy as mutually exclusive. This study raises concerns regarding medical safety. Knowledge gaps among HCPs made participants turn to peers for medical advice. HCPs did not expect lactogenesis after chest masculinization surgery, failed to provide information on nursing and to prevent mastitis. The exclusion of legal males from the EMR limited information transfers between clinics and disabled standard procedures, posing a risk to patient security. The notion that only women get pregnant prevented the timely diagnosis of premature labor. **As noted in a previous study**, gynecological problems will	Positive/Methodologically sound

	experience from the Stockholm gender clinic, performed all interviews. After demographic information on gender identity, access to gender-affirming treatments, age, occupation and civil status had been collected, interviews started with the question: What was it like for you to undergo pregnancy as a man/trans masculine/non-binary individual?<br><br>6. Yes.	the area of study was limited. Initially transcripts were read for an overview. Text units relevant to the aim were then identified, coded and sorted into main- and sub-categories. These were then revised to merge or regroup overlapping categories and identify a common theme. Transcripts were read repeatedly to ensure that no relevant information was misunderstood or lost. Clarifications and excisions (three dots) from quotations are marked with squared brackets.<br><br>9. Yes. <u>Findings</u>:Providers in gender clinics, antenatal care and delivery were perceived to regard a masculine gender identity and pregnancy as incompatible. The main categories encompassed expectations and experiences of pregnancy related care and participant responses to it. Participants took charge of their care to ensure that their needs were fulfilled. The quality of care was inconsistent.	only be identified as the cause of abdominal pain when HCPs perceive patients as female, why pregnancy complications in trans masculine patients risk being misdiagnosed (Stroumsa et al., Citation2019). Gender binary and cisnormative assumptions were thus associated with medical risks.	

**4. Galvão DLS, Araújo WJS, Brandão Neto W, Barros MBSC, Monteiro EMM.**<br><br>**Intersectionality and challenges in support for chest-feeding transgender men.**<br><br>Brazil<br>two bisexual transmen (n=2)	1.Yes. <u>Aim</u>:To, from an intersectional standpoint, grasp the challenges experienced by health professionals and service users of human milk banks in provision of care for transgender men chestfeeding.<br><br>2. Yes<br><br>3.Yes<br><br>4.Yes. <u>Recruitment</u>: Trans men who had chestfed their babies were selected by "snowball" sampling, the first participant being nominated by a specialised LGBT care service. The researcher contacted this trans man via WhatsApp to provide information about the research and, through him, it was possible to nominate another participant, totalling two bisexual trans men.<br><br>5.Yes. <u>Data collection</u>: The interview scripts were used for each group of participants to consider the challenges in caring for trans men in breastfeeding support	7.Yes. <u>Ethical consideration</u>: The interviews began after a declaration of free and informed consent was read, explained and formally consented to, in person or virtually.<br><br>The study was approved by the research ethics committee of the Universidade Federal de Pernambuco under Ethical Appreciation Presentation Certificate No. 58300022.1.0000.5208 and conducted in accordance with the topics of the Consolidated Criteria for Reporting Qualitative Research (COREQ)7.<br><br>8. Yes. <u>Data analysis</u>:The analysis was carried out in pairs following the thematic analysis proposed by Braun and Clarke, for each segment of the group of participants, following the six steps and using Atlas.ti (version 9.0) software as a support tool.<br><br>9. Yes. <u>Findings</u>: Overall, the findings of this study show	10.Yes. <u>Valuability</u>: There are no protocols covering the possible manners of feeding a baby in non-normative families to guide professionals supporting chestfeeding.<br><br>Health professionals occupy a position of power, which is reflected in their relations with trans people in relation to chestfeeding, highlighting the importance of this practice for the child's health, on the public health paradigm, to the detriment of the subjectivity of those who chestfeed. The trans population occupies a place from which to denounce discrimination and voice demands for transcompetent care.<br><br>Intersectionality offers a broader view of these inequities for them to be considered as a whole when implementing public policies for equitable care for the trans population in HMB environments.	Positive/Methodologically sound

	services. Each participant was interviewed once, between August and October 2022. Interviews lasted an average of 30 minutes and, with the prior authorisation of the participants, were recorded and transcribed. Each transcription was then validated by the respective interviewee. After responding to a questionnaire to record their socio-demographic characteristics, participants were interviewed and their physical characteristics were recorded in a field notebook, along with those of the HMB environment.<br><br>6. Yes.	the need to break with breastfeeding-related paradigms shaped by cisheteronormativity.<br><br>Lacunas in the educational, institutional and management spheres, associated with personal and social issues, reproduce a pre-conceived normative model and disregard the special demands of providing chestfeeding care for the trans population. Cisheteronormativity and "professional supremacy" operate in personal, social and institutional respects to segregate transgender men in lactation support services. Intersectional analysis of these challenges affords an overall view of segregative factors and enables public policies to be introduced to promote social justice.	Limitations:This study had limitations. The first was the small sample of health professionals working in HMBs and of trans health service users, which may relate to taboos on the subject of gender identity. The second was that participants were exclusively bisexual, trans men in the context of chestfeeding, while trans women wishing to lactate were not included. The lack of articles on chestfeeding among Brazil's trans population precluded comparisons with the international trans population or with cisgender women in Brazil.	
**5. Goldberg, A. E., Downing, J. B., & Sauck, C. C.**<br><br>**Perceptions of children's parental preferences in lesbian two-mother**	1.Yes. Aim:To explore how lesbian mothers perceive their 3 1/2-year-old children's parental preferences in families in which one mother is genetically linked to the child.	7. Unknowns. Ethical consideration: Interested couples were mailed consent forms assuring confidentiality and detailing the conditions of participation. Women returned the consent formin	10.Yes Valuability: (see "limitations")<br><br>Limitations: From 2008. Outdated idea of the term "biological". The limitations mentioned in the study are	Unknowns<br><br>*No approval has been sought from the ethics committee.*

**households. Journal of Marriage and Family**<br><br>USA, Massachusetts<br>30 same-sex couples - 60 women (n=60)	2. Yes<br><br>3.Yes<br><br>4.Yes. Recruitment: All 30 couples were recruited during their pregnancy. Recruitment efforts were multifold. Study Information was posted in the offices of gynecologists and midwives in Massachusetts and published in gay community newsletters. Calls for participants were posted on national websitesand in newsletters pertaining to lesbian issues to obtain a geographically diverse sample.<br><br>5.Yes. Data collection: Both partners were interviewed individually by telephone when their first child was 3½ years old and were sent questionnaire packets to complete within several weeks of the interview. Womenwere asked to be available at a time and place that would ensure that their conversations were pri-vate. Interviews lasted about 1 – 1.5 hours; the questionnaires	the initial questionnaire packet.<br><br>8. Yes.Data analysis: Grounded theory methods were used in the current analysis. Specifically, we used comparative methods to establish analytic distinctions by comparing data across participants to identify similarities and differences. We began by conducting line-by-line coding, attending closely to participants' interpretations. After we read and coded all the data, we applied the coding scheme to the data, which allowed for both the identification of more descriptive codes and the generation of themes for which there was the most substantiation. We reapplied the scheme and made subsequent revisions until alldata were accounted for.<br><br>9. Yes.Findings:We organize our findings around our final coding scheme, which appears in three sections;	also questionable, based on more updated medical research, such as partner donation and induced lactation.<br><br>Also, most women in the sample are Caucasian and financially secure; the study does not explore how differences in race, ethnicity,or social class may impact women's experiences of child preference.	

	took 30 minutes to complete.Women returned their questionnaires separately from their partners in postage-paid envelopes.<br><br>6. Yes.	- First, we discuss the range of women's percep-tions of (and explanations for) their children's parental preferences.<br>- Second, we discuss wom-en's feelings about such preferences.<br>- Third, we discuss women's strategies for responding to them.		
**6. Hoffkling A, Obedin-Maliver J, Sevelius J.**<br><br>**From erasure to opportunity: a qualitative study of the experiences of transgender men around pregnancy and recommendations for providers.**<br><br>USA/ Western Europe?<br>Ten transgender men (n=10)	1.Yes. Aim:To understand the needs of transgender men who had given birth.<br><br>2. Yes<br><br>3.Yes<br><br>4.Yes. Recruitment:Participants were recruited for interviews from a pool of prior participants of an online convenience sampled survey, conducted in 2013.<br><br>5.Yes. Data collection: Interviews were conducted, over a 2-month period, until theoretical saturation was reached at 10 interviews. Interview order was based on response speed. Interviews	7.Unknown. Ethical consideration: Participants were emailed a consent form 2–4 days before the interview, which was reviewed aloud at the beginning of the interview to obtain verbal consent.Participants were compensated with a $35 Amazon gift card or PayPal payment, according to participant preference. Participants could choose to have their contact information destroyed or retained to facilitate distribution of published study results.<br><br>8. Yes.Data analysis: Interviews were recorded, professionally transcribed,	10.Yes. Valuability: Participants in this study expressed some clear values on research priorities, in particular a consistent aversion to research that sees their existence as something that needs to be explained or justified, e.g., "I'm not interested in 'why some people are born trans and some people are not'"<br><br>The first, and most central, element is that some of the people who need obstetric care are not women.<br><br>Limitations: Because of the small sample size in this study, the fact that not all interviews covered all the topics, and the likely	Unknowns<br><br>*No approval has been sought from the ethics committee.*

	were conducted online, using the video remote-conferencing software program BlueJeans for eight interviews and two were conducting using audio only. The lead author (AH) conducted all interviews.<br><br>6. Yes.	and coded using Dedoose qualitative analysis software. Two transcripts, selected purposively for diversity of content, were coded using a priori codes, and emergent codes were identified and applied. Emergent codes were reviewed by three researchers to produce the final code set, and then all transcripts, including the initial transcripts used for code-development, were (re)coded using the final code set. Two of these transcripts were fully coded by a second researcher, and reviewed by two more, for validation of coding reliability. No major differences of interpretation arose, indicating a high level of coding reliability.<br><br>9. Yes.Findings: Participants reported diverse experiences and values on issues including prioritization and sequencing of transition versus reproduction, empowerment in healthcare, desire for external affirmation of their gender and/or pregnancy, access to social	selection bias due to convenience sampling, we chose not to present any quantitative data such as frequency counts.<br><br>This study was limited to English speakers, and participants were entirely from the US (80%) or Western Europe. Furthermore, because of the original study inclusion criteria, the present study excluded transgender and gender-nonconforming individuals who did not identify as male, as well as those who did not have pregnancies resulting in live birth. We are also missing the experiences of transgender men who chose to never conceive, those who wished to conceive but were not able to, or those who conceived but whose pregnancies ended in miscarriage, abortion, or stillbirth. As other studies have shown, participants engaging in online studies are disproportionately educated and economically secure. Additionally, since	

		supports, and degree of outness as male, transgender, or pregnant. Anticipatory guidance from providers was central in promoting security and empowerment for these individuals as patients.	advertisements for the study were distributed through transgender websites and community centers, our sample may not represent those who are not connected to these services.	
7. **Jackson, J. E., Wild, R., Hallam, J., Graves, R., Woodstein, B. J., & Stothard, P.**<br><br>**Exploring the healthcare experiences and support needs of chestfeeding or breastfeeding for trans and non-binary parents based in the United Kingdom.**<br><br>United Kingdom<br>Seven trans or non-binary participants (n=7)	1.Yes.Aim:To contribute to the recent body of literature exploring the needs of trans and non-binary people during early parenting as a continued effort for addressing health inequity.<br><br>2. Yes<br><br>3.Yes<br><br>4.Yes. Recruitment:Data were collected from 7 participants who responded to a recruitment call shared by trans organizations through Twitter, Facebook and Instagram. An initial phase of deeper outreach to Black and other minoritized trans parents was unsuccessful in recruiting Black or Southeast Asian parents.	7.Yes. Ethical consideration: Before taking part in the study all participants received (i) information about the study that outlined what their involvement entailed, how their data would be stored securely and how their identity would be protected and (ii) a copy of the interview schedule. This information allowed the participant to give informed consent ahead of the interview and provided an opportunity for them to select a pseudonym that is used throughout the analysis. After the interview, all participants were debriefed; as part of this, they were reminded of their right to withdraw and signposted to trans charities that could be contacted if	10.Yes. Valuability: there is a need for HCPs to have the support needed to share evidence-based advice with trans and non-binary parents as this improves health outcomes. As evidenced in the analysis the advice around testosterone and body bodyfeeding given by HCPs was inconsistent and at times contradictory to other evidence. This led to a breakdown for one participant and withdrawal of treatment for another. In both cases there was a sense that there was a lack of trust with the HCP and the participants turned to their community for support, information and reassurance. This indicates that healthcare services need to work more closely with trans people and create easily accessible	Positive/Methodologically sound

	A decision around sample size was made in situ during the project and the volume of data was monitored as it was being collected (Braun & Clarke 2021). In part, the sample size was determined by the practical constraints of working with minority populations in a timescale set by funders. Future research must consider this from the outset.<br><br>5. Yes. <u>Data collection</u>:Data were collected using a semi-structured interview schedule that invited the participant to reflect upon their reasons for choosing to try bodyfeeding. Additionally, their experiences of establishing and continuing their feeding practice with recommendations for other parents seeking to make the same decision were explored. All participants received payment of £10 amazon evoucher as a gesture of thanks for their time and insight.All interviews were dyadic and were conducted	further specialist support was required.<br><br>The study was approved by the University's ethics committee (ref ETH 2122-3794).<br><br>8. Yes.<u>Data analysis</u>:The analysis was guided by Reflexive Thematic Analysis. Given the small, exploratory nature of the project, and a desire to learn from trans and nonbinary people about their experiences, themes were identified using an inductive approach. This requires the analyst to consciously put aside preconceptions of what may be important/present in the data based on personal experience or professional interest and allow the participant's words to guide the analysis as much as possible. The differing insider and outsider identities of the research team and engagement group worked to support the analytic process and the identification of themes using the 6-step	resources that can be utilized by HCPs.<br><br><u>Limitations</u>: Research on trans and non-binary individuals has predominantly focused on white, middle-class, and non-disabled populations, and this is also true of research on trans and non-binary parents. An intersectional framework, where multiple axes of identity are considered in the analysis,<br>may be most useful for future research which explores the experiences of trans and non-binary parents.	

	online using Microsoft Teams by either JJ or RW. There were no significant differences between the types of information shared with the different researchers. This is noteworthy given the insider status of RW and the outsider status of JJ. The interviews were transcribed verbatim (with any identifying information removed or changed) for analysis.<br><br>6. Yes.	process outlined by Braun and Clarke<br><br>9. Yes.<u>Findings</u>: the analysis identified three themes which focused on (i) the baby and doing the right thing for them, (ii) the experiences of a changing body and the support desired whilst navigating this, and (iii) the experiences the parents faced with HCPs throughout their feeding journey		
**8. Juntereal NA, Spatz DL.**<br><br>**Breastfeeding experiences of same-sex mothers.**<br><br>USA, Pennsylvania Eighteen gestational mothers (n=18)	1.Yes. <u>Aim</u>:To explore the lactation experience and level of lactation support of birth mothers in a same-sex (two female) relationship.<br><br>2. Yes<br><br>3.Yes. This study used a mixed methods design.<br><br>4.Yes. <u>Recruitment</u>:The online survey was deployed nationally through email, word of mouth, and online platforms, primarily Facebook. Four interview	7.Yes. <u>Ethical consideration</u>: For the qualitative arm, informed consent was obtained from all participants before interviewing.<br><br>The study was reviewed by the University of Pennsylvania Institutional Review Board and deemed exempt. All data used for analyses were deidentified and coded with pseudonyms the participants chose for identity protection. All data for this study were stored on	10.Yes. <u>Valuability</u>: Same-sex mothers can equally contribute to the breastfeed-ing role. These mothers recognize human milk as the optimal enteral nutrition for infants and value the mother-child bond inherent to breastfeeding. Health care practitioners must bet-ter educate themselves to support their breastfeeding goals. Professional organizations must update their position statements on breastfeeding to include LGBTQ parents. Including	Positive/Methodologically sound

	informants contributed to the survey dissemination by posting a recruitment flyer to their online networks. Interested participants contacted the principal investigator via email.<br><br>5.Yes. <u>Data collection</u>: Qualitative interviews were conducted 1-2 weeks after first contact. The first author scheduled and conducted all informant interviews. Interviews were scheduled at a mu-tually agreeable time and were conducted either in person or via telephone for 1 hour at maximum.<br><br>6. Yes.	a password-protected secure network drive.<br><br>8. Yes.<u>Data analysis</u>: A semi-structured interview guide which consisted of open-ended questions with probes was used. Open-ended questions allow the participants to narrate their experience without limitation. The guide and probes were accordingly modified to each informant's responses. All interviews were digitally audio-recorded and checked for accuracy by both authors. The Interview data were then uploaded to NVivo qualitative data analysis software (QSR International Pty Ltd. version 12, 2019) for qualitative data management. The first author coded the data and compiled the first-level codes into categories. Together, the principal investigator and first author condensed the categories and grouped them into themes. The identified themes were shared to the last participant and a previous participant as a member check to determine that the final themes remain	language such as “parent,” “same-sex,” and “queer” throughout their position statements and section on induced lactation will guide health care practitioners to be more attentive to asking and referring lactation information to these growing families.<br><br><u>Limitations</u>: As with any research, there are limitations to the study. Participants who answered the survey may have been morewilling to share their personal breastfeeding experiences.Those women who participated in qualitative interviews mayhave also had different experiences than women who choose not to participate. The authors did not include demographic questions about income level, social class, level of education,or the exact sexual orientation of the participants. This is a limitation of our research and should be explored in future studies	

		salient to the informants' experiences and responses.<br><br>9. Yes.Findings:<br>Eighteen gestational mothers participated in qualitative interviews. Five themes emerged from the qualitative data:<br>(a) committed to a year<br>(b) deciding “how to do it,”<br>(c) sources of information<br>(d) involvement of partner<br>(e) need for inclusive and educated health care practitioners.		
**9. Klittmark S, Niit JKP, Nerström E, Grundström H, Nieminen K, Wells MB, Malmquist A.**<br><br>**LBTQ parents' needs for support postpartum following a complicated birth: A matter of reproductive justice.**<br><br>Sweden<br>22 participants (n=22)	1.Yes. Aim:To explore reproductive injustices in postpartum care for LBTQ people.<br><br>2. Unknown.<br><br>3.Yes<br><br>4.Yes. Recruitment: 22 participants were recruited through advertising in different Facebook groups for LBTQ parents in Sweden (n = 9), emailing participants in an antenatal education program for LGBTQI parents	7.Unknown. Ethical consideration: Participants received oral and written information about the study and provided their written informed consent to participate. The interviews were audio recorded and transcribed verbatim except for pseudonyms replacing names of participants.<br><br>The research project has been approved by the Swedish Ethical Review Board (2021-05801-01). The first author worked as a midwife in a parental support	10. Yes.Valuability:The results further show the need for validation of the non-birth parent and inclusive breast/chest-feeding support. Results emphasize the need for more psychosocial support around the birth experience, including better medical support and information during the whole process of childbirth.	Positive/Methodologically sound

	(n = 8), and by snowball recruitment (n = 5).<br><br>The inclusion criteria were self-identified LBTQ birth or non-birth parents who had experienced obstetrical and/or neonatal complications when their child was born.<br><br>5.Yes. Data collection: The current study uses a qualitative cross-sectional interview methodology.<br><br>6. Yes.	program for LGBTQI prospective parents and had cared for some of the participants. These participants were interviewed by other researchers.<br><br>8. Yes.Data analysis:The authors used Systematic Text Condensation [STC] by Malterud to organize, interpret and summarize the data. STC uses four steps to identify units in the text that make up categories which are used to reorganize the text in a meaningful and transparent way.<br><br>9. Yes.Findings:Results point to the importance of providing an LBTQ safe space, which includes the need to feel safe regarding one's gender or sexual identity, by avoiding cisheteronormative assumptions and using inclusive language. In the context of recently experiencing birth complications, parents needed a space where they were able to focus on physical and mental healing.		

**10. MacDonald T, Noel-Weiss J, West D, Walks M, Biener M, Kibbe A, Myler E.**<br><br>**Transmasculine individuals' experiences with lactation, chestfeeding, and gender identity: a qualitative study.**<br><br>Canada<br>22 participants (n=22)	1.Yes. Aim:To answer an open research question, “What are the experiences of transmasculine individuals with pregnancy, birthing, and feeding their newborns?”<br><br>2. Yes<br><br>3.Yes<br><br>4.Yes. Recruitment:A convenience sample of participants was recruited mostly through the internet. A letter and a poster were used to recruit participants. The letter was posted to the Facebook page, “Birthing and Breast or Chestfeeding Trans People and Allies,” (formerly “Birthing and Breastfeeding Transmen and Allies”) managed by the first author (TMD). The poster was circulated through email.<br><br>Inclusion criteria for the main study specified individuals who self-identified as transmasculine regardless of stage of transition. Participants were required to have experienced or to be currently experiencing	7. Yes. Ethical consideration: To allow for personal preference and to ensure convenience, safety, privacy, and confidentiality, each participant chose the time of their interview and whether it would be a telephone call or a Skype call with cameras. All interviews were audiotaped using a digital recorder, and then transcribed verbatim, de-identified, and shared with the team to consider themes that emerged as the interviews were conducted. Interviews continued until it was determined we had a wide variety of experiences with consistent, common topics.<br><br>This study was approved by the Health Sciences and Science Research Ethics Board at the University of Ottawa, Ontario, Canada. The approval is based on the Tri-Council Policy Statement: Ethical Conduct for Research Involving Humans (2nd edition). Participants provided informed consent in writing or verbally on a digital	10. Yes.Valuability:The information gathered in this study sheds light on a wide range of transmasculine individuals’ experiences with lactation and infant feeding. Study participants shared stories that were personal and nuanced. There is no single experience that is the transgender experience of infant feeding. It is apparent that transmasculine individuals who give birth sometimes choose to chestfeed, even when they have had previous chest surgery, while others do not chestfeed their babies for physical and mental health reasons. Care providers should communicate an understanding of gender dysphoria and transgender identities in order to build patient trust and provide transgender-competent care. Further, HCPs need to be knowledgeable about lactation and chest care during binding and following chest masculinization surgery, regardless of the chosen feeding method, through all stages: before	Positive/Methodologically sound

	pregnancy and birth, and there was no requirement regarding type of infant feeding. Inclusion was not restricted by geography, but all interviews were conducted in English.<br><br>5.Yes. Data collection: TMD used a questionnaire to collect demographic and descriptive information about participants before each interview. He then used a semi-structured interview guide that included a list of open-ended questions and prompts. The questionnaire used to collect demographics information and the semi-structured question guide used as a basis for interviews are included in Additional file 1. Interviews were conducted by Skype or telephone.<br><br>6. Yes.	recording after having opportunities to ask questions about the research.<br><br>8. Yes.Data analysis:The transcribed interviews were uploaded to NVivo 10 and JN-W, MW, and TMD coded data. We used a combination of line-by-line coding and overall analyses to identify associations, patterns, and relationships. These steps were guided by qualitative description analyses methods. All interviews were coded by at least two researchers. Each used their own topics and then topics were merged. The topics that emerged from the data about infant feeding were well-defined and practical. After the topics had been identified from the interviews, we reversed the process and the sets of approved quotes were recoded under the topics to ensure we had not missed any key topics.<br><br>Methodologically, we based our analysis on interpretive description analysis. We	pregnancy, pregnancy, and post birth.<br><br>Limitations: The key limitation for this study is about transferability. It is unknown whether the convenience sample recruited reflects the general population of transmasculine individuals experiencing pregnancies. All of the study participants were university educated, and the majority were financially secure and in a committed relationship.<br><br>Further studies on this topic should attempt to include more low-income individuals and those with less formal education, and they should also aim to better understand geographical considerations. Future research could try to determine the effect of different types of chest masculinization surgery on lactation. As a possible trend to investigate, it was unknown that the two participants with previous chest masculinization surgery who had significant mammary tissue re-growth	

		wanted an analysis that would provide a rich description while at the same time giving us an interpretation that would lead to recommendations for transmasculine individuals considering pregnancy or chestfeeding and for HCPs who work with transgender patients and clients.<br><br>9. Yes.<u>Findings</u>: The participants' experiences of gender dysphoria, chest masculinization surgery before pregnancy or after weaning, accessing lactation care as a transmasculine person, and the question of restarting testosterone emerged as data.	and some amount of lactation following birth both had surgeries that preserved the nipple stalks instead of surgeries involving nipple grafts. The experiences and needs of transmasculine individuals who had unplanned pregnancies could be an important topic for future research.	
**11. Melo, F. E. B. and F. B. Scagliusi (2026). "Lactation induction, shared breastfeeding, and the right to opacity: a reflection on the use of health protocols and practices for women who have**	**Section A - Are the results valid?**<br><br>1. Yes. <u>Aim</u>: This article is derived from a qualitative research study, whose general objective was to narrate and analyze how couples of cisgender women perceive and give meaning to the experience of double breastfeeding, with the	**Section B: What are the results?**<br><br>7.Yes. <u>Ethical considerations</u>: This study was submitted to and approved by the Ethics Committee of the Faculdade de Saúde Pública, Universidade de São Paulo under the CAAE:	**Section C: Will the results help locally?**<br><br>10. Yes. <u>Valuability</u>: it is not necessary to understand the other – or their life logics – in order to form a care alliance that goes beyond rigid, standardized protocols.<br><br>*"Moreover, we propose that the right to opacity become*	**APPRAISAL SUMMARY:**<br><br><br>Positive/Methodologically sound

	**relationships with women." Einstein (Sao Paulo) 24(spe1): eRW2297. https://doi.org/10.31744/einstein_journal/2026RW2297**<br><br>Sao Paolo, Rio De Janeiro, Brazil<br>Seven cisgender white women who have relationships with women (n=7)	analysis based on feminist authors from the Global South, in line with a process of constant positionality, reflecting the negotiations that occur in the field and an understanding of the complexity of the ethical commitment of social research with vulnerable participants.<br><br>2. Yes.<br><br>3. Yes.<br><br>4. Yes. Recruitment:a search was conducted on social media using suitable hashtags. After identifying potential participants, initial contact was made. Additionally, key contacts, built throughout previous research on a related topic, were activated to help publicize the project. After recruiting the first participants, the snowball method was adopted to increase the sample.<br><br>5. Yes. Data collection: For data production, semi-structured interviews were	6141523.8.0000.5421; #6.598.184.<br><br>8. Yes. Data Analysis: To begin the analysis, a broad research<br>Question was proposed: What is the experience of following lactation induction protocols for cisgender women who opted for double breastfeeding? To answer this question, a thematic content analysis was used (Burnard). From there, open coding of the data was initiated, whilst reflecting on the structures on which the ontoepistemological assumptions that guide and particularize the research process are founded.<br><br>9. Yes. Findings: From this analysis, two analytical categories emerged: the first addresses the sources of information used by the participants, highlighting the key role of information networks built through the internet and the notable absence of healthcare professionals as references	*one of the horizons for the construction of practices within the field of health, not only in clinical practice, but also in scientific production."*	

	conducted – between June and October 2024 – via video calls lasting an average of 40 minutes.<br><br>Has the relationship between researcher and participants been adequately considered?<br><br>6. Yes.	in this process. The second category discusses the participants' experiences with the induction protocol, highlighting the challenges of balancing their routines with the protocol's demands and the importance of healthcare professionals who act as positive deviants from the hegemonic model of care.		
**12. Pelka S.**<br><br>**Sharing motherhood: maternal jealousy among lesbian co-mothers**<br><br>California, USA<br>30 lesbian couples<br>(n=60 participants)	1. Yes. <u>Aim</u>: The following article focuses on how variables common to lesbian-led families, such as infant preference, infertility, and gender and role identifications, correlate with feelings of jealousy between lesbian co-parents. The experience of feeling jealous of one's partners' relationship with one's child is important to explore in order to provide lesbian co-mothers a reference point to validate their own feelings. It also provides information that may help professionals serving lesbian-led families better understand and support their unique family configurations.<br><br>2. Yes.	7. Yes. <u>Ethical considerations</u>: The research was approved by the Institutional Review Board at The University of Chicago and all research informants gave informed consent to participate.<br><br>8. Yes. <u>Data analysis</u>: Interviews were tape recorded, transcribed, and analyzed using grounded theory emphasizing an "emergent approach" of consistent, comparative analysis.<br><br>Transcripts were content coded to recognize different themes that emerged in informant narratives. Approximately one year after	10. Yes. <u>Valuability</u>: Therapists and professionals that serve lesbian and gay co-parents should be sensitive to the unique emotional needs of variously created families, particularly when only one partner is biologically related to their children.<br><br><u>Limitations</u>: The generalizability of the findings may be limited by the size and composition of the sample. To some "happy" couples that felt confident enough in their personal relationships and sexual identity to be interviewed and observed at length. The vast majority of informants were known as lesbians, extent,	Positive/Methodologically sound

	3. Yes.<br><br>4. Yes. Recruitment: Because lesbian parenting couples are difficult to locate, nonrandom sampling techniques were used to recruit study informants. Informants were recruited through a gay and lesbian parents' listserv, an advertisement placed in a lesbian-oriented magazine, a virtual message board for women using IVF, a local lesbian parent support group, and word-of-mouth.<br><br>5. Yes. Data collection: Findings are based on in-depth interviews conducted between 2001 and 2004 with a total of 30 lesbian couples (N = 60 women) with at least one child. The vast majority of informants live in a major northeastern coast city. For purposes of broadly comparing the influence of a perceived biological connection of parents to their children, three ostensibly different sample populations of lesbian-led families were	being interviewed, informants were mailed a self-administered follow-up questionnaire in order to get clarification on missing data from the interview. There was a 60% response rate with equal number of returns from all three samples. The researcher also periodically checked in with informants by e-mail or phone to clarify an interpretation of narrative data or to gather missing data.<br><br>9. Yes. Findings: The first major finding is that it is possible for lesbian couples to experience MJ. Factors that appear to contribute to higher probabilities of MJ include: dual desire to carry a pregnancy (a variable which is conflated in this study to some degree with having "role similarity"); the experience of infertility (conflates with "desiring to be pregnant"); and perceived unequal biological ties of mothers to their children. Infant preference behaviors do not appear to be an independent predictor of	study informants were a self-selecting group. They were relatively both to their wider families and in much of their professional lives. In this way, informants may be qualitatively different from other lesbian parenting couples that do not share these qualities. The fact that even relatively "happy" couples reported struggles with jealousy suggests that this experience may be underrepresented in the lesbian, gay, and other parenting communities in which there are asymmetrical biological ties of children and their parents. Generalizability is further limited by race and socioeconomic status as the majority of informants were highly educated women of Caucasian descent earning professional incomes.<br><br>If infertile or unwilling to be pregnant, economically poorer lesbians are less likely to find a way to have children.	

	recruited for study: Ten couples had adopted infants, 10 couples created their families using AI, and 10 couples used IVF.<br><br>The average interview lasted three hours and took place with both partners present. Not only did informants appear more relaxed in joint interviews, but they also often challenged each other when they felt that their partner wasn't being forthcoming on a particular issue. Joint interviews gave the researcher insight into how the women related to one another as a couple.<br><br>6. Yes.	maternal jealousy as IP was only reported to be difficult for couples in which both women desired to be birth mothers. Conversely, this study suggests that the perception of equal biological relationships of both mothers to the child (as in the case of elective IVF and adoption) or being a couple in which only one partner desires to carry a pregnancy are factors that often protect against MJ. Findings suggest that lesbian couples that pursue their ideal families through AI and IVF should be prepared for the experience of infant preference for the birth mother, particularly if she breastfeeds.		
**13. Pereira DMR, Peçanha LMB, Araújo EC, Sousa AR, Galvão DLS, Lima MFG, Souza MC, Torres KCC.**<br><br>**Experiences and meanings of trans men about breastfeeding in light of the Theory of Social Representations.**	1.Yes. <u>Aim</u>: To analyze the experiences and meanings attributed by trans men to the breastfeeding process in light of the Theory of Social Representations.<br><br>2. Yes<br><br>3.Yes	7.Yes. <u>Ethical consideration</u>: This study was approved by the Federal University of Pernambuco (UFPE) Research Ethics Committee, under<br>Opinion 4.862.503, approved in 2021, in accordance with Resolution 466/12 of the Brazilian National Health Council (BNHC). Participants were informed about the	10. Yes.<u>Valuability</u>:This study<br>may promote reflections for changing nursing and health practices in different care settings, especially during prenatal care, childbirth and the postpartum period.<br><br><u>Limitations</u>: This study has limitations, since data collection was carried out via	Positive/Methodologically sound

Brazil<br>Five bi/hetero/pan sexual transgender men (n=5)	4.Yes. Recruitment:The current study was conducted with participants associated with prominent organizations dedicated to the LGBT community. The institutions included were the Brazilian National LGBTI+ Alliance, the Patrícia LGBT Outpatient Clinic Gomes, located in Recife, and the LGBT Comprehensive Health Care Coordination of the state of Pernambuco. The choice of these locations took into account the importance of gathering and recognizing this population of interest, meeting the necessary criteria to compose the sample and conduct the research.<br><br>The intentional sample for this study consisted of five transmen, after including trans men who experienced breastfeeding and were 18 years of age or older. Trans men who experienced breastfeeding with cognitive dysfunctions that would hinder participation in the study, who did not have technological resources	potential risks, benefits and procedures involved in the study, as described in the Informed Consent Form (FICF) and the Image and Testimonial<br>Release and Consent Form, which were made available online through Google Forms®<br>. All information that could identify participants was anonymized, using self-declared pseudonyms at the time of data collection, thus ensuring that participation in the study was completely voluntary and spontaneous.<br><br>8. Yes.Data analysis:The content analysis technique was applied, aiming to analyze the data, which organizes information into pre analysis, material exploration, treatment of results, inference and interpretation. Data analysis was anchored by the TSR of Serge Moscovici and followers, as it offers a theoretical basis<br>for understanding the everyday life of specific social groups.	Google Meet® as a strategy to prevent COVID-19, which made it impossible to observe the daily lives of participants.	

	(computer, cell phone, tablet, internet, among others), making it impossible to carry out the research in the context of the pandemic, were excluded. The selection of trans men was based on criteria of convenience and availability. The choice for this approach was motivated by the need to engage the population of interest, since this is a group that is difficult to identify.<br><br>5.Yes. Data collection: Data collection took place from September to October 2021, using the technique of individual interviews with a semistructured script, virtually, via Google Meet®. An objective questionnaire was also applied to characterize the sample. The use of the semi-structured interview technique allowed for a more comprehensive acquisition of information, since it allowed the formulation of new questions based on participants' responses. This had a positive impact on empirical data collection for this study.	9. Yes.Findings:Trans men in labor were willing to breastfeed their child, anchoring their representations of breastfeeding in the idea that it is a phenomenon performed exclusively by women and also recognizing this phenomenon as an event that constitutes their masculinity, in addition to describing it as a pedagogical and learning experience with the objective of guaranteeing the healthy development of children.		

	6. Yes.			
**14. Rippey, P. L. F., & Falconi, L.**<br><br>**A land of milk and honey? Breastfeeding and identity in lesbian families.**<br><br>Canada<br>four couples and two birth mothers (N=10 participants)	1.Yes. <u>Aim</u>: To explores the relationship between breastfeeding and identity in lesbian-identified families.<br><br>2. Yes<br><br>3.Yes<br><br>4.Yes. <u>Recruitment</u>: The respondents were found through word of mouth, snow-balling, and a poster that was distributed via email seeking participants in Canada and the United States. A number of the respondents are friends with each other, although they do not all necessarily live near one another.<br><br>All of the respondents were openly and confidently self-identified as lesbian, and with some variation, they all self-identified as mothers and as women; although one birth mother and three non-birth mothers in one way or another suggested that they occupied space between	7.Yes. <u>Ethical consideration</u>: Ethics approval was received through the lead author's university at the time of the interviews and all respondents' names have been changed to protect their confidentiality. We are also careful in our analysis not to link too many identifying characteristics to the respondents within our results, to ensure that respondents' confidentiality can be maintained.<br><br>8. Yes.<u>Data analysis</u>:For the qualitative interviews, we used a theoretical thematic analysis by identifying the important themes that emerged from the research within the context of past theory, rather than a purely inductive approach. To carry this out, all but two of the interviews were transcribed into a single Microsoft Word file for coding. A former graduate assistant of the PI transcribed all but one of the interviews; the final interview	10. Yes.<u>Valuability</u>: The human race has been sustained for millennia by women's breasts nourishing human life, and yet, both the power of this act and the burdens it produces are most often taken for granted. Exploring breastfeeding within lesbian-parent families challenges tacit assumptions and has the potential to deepen our understandings of not only infant feeding, but of how gender and sexual orientation are inextricably connected in their constructions.<br><br><u>Limitations</u>: Although the homogeneity of the sample allows for the identification of differences due to breastfeeding, the homogeneity is also a weakness. The sample should not be viewed as a representative sample of any particular group including Americans, Canadians, lesbians, mothers, or breast-feeders. Perhaps more	Positive/Methodologically sound

	male and female or mother and father, but still referred to themselves as mothers, lesbians, and women. Most had been out since their late teens or early twenties and were in committed partnerships for between 8 and 10 years; all were legally partnered or married.<br><br>5.Yes. Data collection: This research is drawn from a pilot-study of qualitative interviews and a short survey of four couples plus two birth mothers, thus incorporating information from six families. The interviews were carried out between November 2011 and April 2012 over the phone and in person. All but one of the interviews involved interviewing the partners separately and all but two were audio-recorded. One couple was interviewed together at the same time and was video recorded to ensure correct attribution to each voice during transcription; and, unfortunately, due to technical difficulties, the audio recording of the first	was transcribed by the co-author.<br><br>9. Yes.Findings: Breastfeeding has a complex role to play within the construction of one's identity as a mother, of one's gender, and of one's sexual orientation. These women's experiences offer interesting insights into the nebulous boundaries of biology and the ways in which taken-for-granted assumptions about what is "natural" are actually social and cultural constructions.	similarities in the breastfeeding experiences would become evident, if there were greater differences in the demographic make-up of the respondents. Due to the planning and cost involved, lesbians who are able to conceive a child tend to have more money, be older, and more established in their careers than heterosexuals who require far less planning to make a baby. Moore (2011) suggests that many studies of lesbians are biased in favor of higher class, white women due to the desire to incorporate two birth mothers. Particularly missing from this study is the role of racial identity with infant feeding, considering that past research has shown clear differences in the meaning attributed to breastfeeding given one's racial background (Blum, 1999; Hausman, 2003).	

	couple interviewed over the phone was unintelligible.<br><br>6. Yes.			
**15. van Amesfoort JE, van Rooij FB, Painter RC, Valkenburg-van den Berg AW, Kreukels BPC, Steensma TD, Huirne JAF, de Groot CJM, Van Mello NM.**<br><br>**The barriers and needs of transgender men in pregnancy and childbirth: A qualitative interview study.**<br><br>The Netherlands<br>Five participants (n=5)	1.Yes. Aim:To explore the needs and barriers of transgender men in family planning, pregnancy, childbirth, puerperium and perinatal care, to improve perinatal care for transgender individuals and their health care providers.<br><br>2. Yes<br><br>3.Yes<br><br>4. Yes. Recruitment: we aimed to recruit all individuals known to have been pregnant and that visited the Center of Expertise on Gender Dysphoria at the Amsterdam University Medical Centers, the primary and biggest expertise center for<br>gender dysphoria in the Netherlands. As incidence of eligible individuals was very low, they were well known and identified by members of the clinical team. For this first	7. Yes. Ethical consideration: Ethical approval for this study was obtained from the institutional review board at the Amsterdam University Medical Centers, location VUmc in Amsterdam, prior to participant recruitment.<br><br>This study was classified by the institutional review board at the Amsterdam University Medical Centers, location VUmc in Amsterdam, as not requiring ethical approval in accordance to the Dutch Act on Medical Research Involving Human Subjects (WMO). Ethical approval number: 2020.354<br><br>This study has been carried out in accordance with the Declaration of Helsinki.<br><br>All study participants provided verbal and written informed consent for this study.	10. Yes.Valuability:The findings help strengthen the foundation of insight in the needs and hurdles of TGI pursuing pregnancy and therefore may guide HCPs to provide equitable perinatal care, and emphasize the necessity of patient-centred gender-inclusive perinatal care.<br><br>Though research and information in regard to perinatal care for TGI remains scarce, we advise to develop a national guideline in regard to perinatal care for TGI and that HCP resort to gender expertise centers for questions and advise for gender-inclusive perinatal care. This will provide the opportunity for high quality and gender-inclusive care, that will benefit the mental and physical health of TGI and their offspring.	Positive/Methodologically sound

	qualitative assessment of transmasculine pregnancy there within our center there was no public recruitement performed.<br><br>Individuals were eligible for the study if they had been pregnant after social transitioning, and had had a live birth in the past 5 years. Further eligibility criteria included: female sex assignment at birth, gender identification on the transmasculine spectrum prior to pregnancy, ≥18 years of age during the study, and language proficiency of Dutch sufficient for an in-depth interview.<br><br>5.Yes. Data collection: We used semi-structured interviews for data collection to allow detailed exploration of participants' experiences regarding family planning,	8. Yes.Data analysis:Interviews were coded using the qualitative analysis software Atlas.ti. Since information on this subject is scarce, grounded theory methodology was employed and an inductive approach was used to find patterns and collect data from the participants' narratives. A constant comparative method was used to analyse interview transcripts. Firstly, the primary author used open coding to code all transcripts chronologically on interview date. When new codes emerged after coding of every subsequent interview, previous coded transcripts were reread and recoded when necessary. After open coding axial coding was applied to identify connections between codes and determine broader categories. Finally selective coding was applied to identify subthemes among the codes and capture the essence of our study. After finishing coding all interviews, two interviews	Limitations: Since in the Netherlands up until 2014 infertility was required prior to legal transitioning, the prevalence of pregnancy in TGI is rising, but the number of TGI who have been pregnant is still very limited. Because the experiences of TGI in regard to pregnancy and the puerperium in the Netherlands were not investigated before, we performed an exploratory study in which we solely recruited TGI that visited the CEGD at the Amsterdam University Medical Centers and subsequently became pregnant. Therefore the population may not be completely representative for other clinics and our study may not represent the entire population of Dutch TGI who have carried a pregnancy and gave birth to a child. Future research should make an effort to include the general Dutch population of TGI, for instance by distributing the call for participants to the target population through flyers or social media, and	

	pregnancy, childbirth, puerperium and perinatal care.<br>All interviews were conducted during the global COVID-19 pandemic. Hence, all interviews but one, were conducted through the remote-conferencing software program ZOOM by the primary author. One physical interview was conducted, but COVID-19 related restrictions were guaranteed.<br><br>6. Yes.	were checked by a second researcher to ensure consistency. The differences that arose were discussed and codes were reassigned by consensus. The final code set that emerged was applied to all transcripts.<br><br>9. Yes.Findings:<br>- Pregnancy is not only limited to cisgender women.<br>- Transgender and gender diverse individuals may choose to (temporarily) refrain from gender affirming therapy in order to become pregnant.<br>- Pregnancy in transgender and gender diverse individuals may enhance feelings of gender dysphoria and isolation.<br>- Health care providers are perceived as unaccustomed and lack adequate tools to provide perinatal care in transgender and gender diverse individuals.<br>- There is a need for patient-centred gender-inclusive perinatal care.	collaborating with other health care providers.	
**16. Ziegler, E., et al. (2026). ""They**	**Section A - Are the results valid?**	**Section B: What are the results?**	**Section C: Will the results help locally?**	**APPRAISAL SUMMARY:**

**Didn't Know How to Support Me": The Experiences of Transgender Men and Non-Binary People With Chestfeeding." J Hum Lact 42(1): 94–104. https://doi.org/10.1177/08903344251401916**<br><br>Ontario, Canada<br>Eight transgender and non binary, semi-structured (n=8)	1. Yes. Aim: Guided by queer phenomenology, the purpose of this study was to explore the experiences of chestfeeding from the perspectives of transgender men and non-binary people.<br><br>2. Yes.<br><br>3. Yes.<br><br>4. Yes. Recruitment: The target population of TNB people who had experience with chestfeeding were recruited through various channels, including online, through word-of-mouth, and from organizations who work with this population. Eligibility criteria included self-identification as TNB, having delivered a baby within the last 5 years, and having attempted to chestfeed or lactate for any length of time. Purposive and snowball sampling was used.<br><br>5. Yes. Data collection: Data were collected between July 2022 and February 2023.	7. Yes. Ethical consideration: Before analyzing the qualitative data, we engaged in a deliberate and iterative reflexivity process to critically examine how our individual identities, professional roles, and prior experiences might shape our interpretations and influence the research design and outcomes<br><br>This study was approved by Research Ethics Boards at TorontoMetropolitan University and McMaster University.<br><br>8. Yes. Data analysis: Data were analyzed concurrently with data collection. Transcripts were coded independently by two or more members of the research team, and then discussed collectively with the full research team. Codes were then sorted into themes and any overlapping themes were consolidated, ensuring they were aligned with the research question. A	10. Yes. Valuability: This study suggests there is a need for inclusive policies, comprehensive provider education, and practices that affirm diverse gender identities across the perinatal period.	Positive/Methodologically sound

	Participants were provided with the consent form in advance.<br>Consent was reviewed prior to the interview to allow the participant to ask any further questions. Three research team<br>members conducted the interviews. All interviews were conducted via secure Zoom teleconferencing, transcribed verbatim, and stored securely as per university policies. Semi-structured interviews lasted an average of 33 minutes.<br><br>6. Yes.	stepwise constant comparative approach was used to assess commonalities and patterns of human experience within and across cases.<br><br>9. Yes. Findings:<br>Transgender men and non-binary people identified a variety of facilitators and barriers to chestfeeding. Having access to support systems, knowledgeable practitioners who provide gender-affirming care, and bodily autonomy were protective factors through the chestfeeding experience. In addition, planning and preparing for chestfeeding experiences added to positive experiences with chestfeeding. Gender dysphoria and transphobic societal norms were barriers to chestfeeding.		

CASP Checklist: CASP Qualitative Studies Checklist

CASP
Critical Appraisal
Skills Programme

CASP Checklist:
For Qualitative Research

Reviewer Name:	
Paper Title:	
Author:	
Web Link:	
Appraisal Date:	

During critical appraisal, never make assumptions about what the researchers have done. If it is not possible to tell, use the "Can't tell" response box. If you can't tell, at best it means the researchers have not been explicit or transparent, but at worst it could mean the researchers have not undertaken a particular task or process. Once you've finished the critical appraisal, if there are a large number of "Can't tell" responses, consider whether the findings of the study are trustworthy and interpret the results with caution.

Section A Are the results valid?

<table>
<tr><td>1. Was there a clear statement of the aims of the research?</td><td>☐Yes ☐ No ☐ Can't Tell</td></tr>
<tr><td colspan="2">CONSIDER:
● what was the goal of the research?
● why was it thought important?
● its relevance</td></tr>
<tr><td>2. Is a qualitative methodology appropriate?</td><td>☐Yes ☐ No ☐ Can't Tell</td></tr>
<tr><td colspan="2">CONSIDER:
• If the research seeks to interpret or illuminate the actions and/or subjective experiences of research participants
• Is qualitative research the right methodology for addressing the research goal?</td></tr>
<tr><td>3. Was the research design appropriate to address the aims of the research?</td><td>☐Yes ☐ No ☐ Can't Tell</td></tr>
</table>

CONSIDER:<br>● *if the researcher has justified the research design (e.g., have they discussed how they decided which method to use)*	
4. Was the recruitment strategy appropriate to the aims of the research?	☐Yes ☐ No ☐ Can't Tell
CONSIDER:<br>● *If the researcher has explained how the participants were selected*<br>● *If they explained why the participants they selected were the most appropriate to provide access to the type of knowledge sought by the study*<br>● *If there are any discussions around recruitment (e.g. why some people chose not to take part)*	
5. Was the data collected in a way that addressed the research issue?	☐Yes ☐ No ☐ Can't Tell
CONSIDER:	

• *If the setting for the data collection was justified*<br>• *If it is clear how data were collected (e.g. focus group, semi-structured interview etc.)*<br>• *If the researcher has justified the methods chosen*<br>• *If the researcher has made the methods explicit (e.g. for interview method, is there an indication of how interviews are conducted, or did they use a topic guide)*<br>• *If methods were modified during the study. If so, has the researcher explained how and why*<br>• *If the form of data is clear (e.g. tape recordings, video material, notes etc.)*<br>• *If the researcher has discussed saturation of data*	
6. Has the relationship between researcher and participants been adequately considered?	☐Yes ☐ No ☐ Can't Tell
CONSIDER:<br>• *If the researcher critically examined their own role, potential bias and influence during (a) formulation of the research questions (b) data collection, including sample recruitment and choice of location*<br>• *How the researcher responded to events during the study and whether they considered the implications of any changes in the research design*	
Section B: What are the results?	
7. Have ethical issues been taken into consideration?	☐Yes ☐ No ☐ Can't Tell

CONSIDER:

- *If there are sufficient details of how the research was explained to participants for the reader to assess whether ethical standards were maintained*
- *If the researcher has discussed issues raised by the study (e.g. issues around informed consent or confidentiality or how they have handled the effects of the study on the participants during and after the study)*
- *If approval has been sought from the ethics committee*

8. Was the data analysis sufficiently rigorous?	☐Yes ☐ No ☐ Can't Tell

CONSIDER:

- *If there is an in-depth description of the analysis process*
- *If thematic analysis is used. If so, is it clear how the categories/themes were derived from the data*
- *Whether the researcher explains how the data presented were selected from the original sample to demonstrate the analysis process*
- *If sufficient data are presented to support the findings*
- *To what extent contradictory data are taken into account*

• *Whether the researcher critically examined their own role, potential bias and influence during analysis and selection of data for presentation*	
9. Is there a clear statement of findings?	☐Yes ☐ No ☐ Can't Tell
CONSIDER:<br>● *If the findings are explicit*<br>● *If there is adequate discussion of the evidence both for and against the researcher's arguments*<br>● *If the researcher has discussed the credibility of their findings (e.g. triangulation, respondent validation, more than one analyst)*<br>● *If the findings are discussed in relation to the original research question*	
Section C: Will the results help locally?	
10. How valuable is the research?	☐Yes ☐ No ☐ Can't Tell
CONSIDER:	

• *If the researcher discusses the contribution the study makes to existing knowledge or understanding (e.g., do they consider the findings in relation to current practice or policy, or relevant research-based literature*<br>• *If they identify new areas where research is necessary*<br>• *If the researchers have discussed whether or how the findings can be transferred to other populations or considered other ways the research may be used*

APPRAISAL SUMMARY: *List key points from your critical appraisal that need to be considered when assessing the validity of the results and their usefulness in decision-making.*		
Positive/Methodologically sound	Negative/Relatively poor methodology	Unknowns

Creative Commons

©CASP this work is licensed under the Creative Commons Attribution – Non-Commercial- Share A like. To view a copy of this licence, visit https://creativecommons.org/licenses/by-nc-sa/4.0/

Supplementary file 5
Analytical procedure, phase 4.

Key phrases: Identity markers, Bonding, Gender Roles, Lack of knowledge, Lack of provider support, Prejudism, Compensating resources and Sacrifice and inner conflict.

(X) = General finding on postpartum experiences among sexual and gender minorities, extending beyond lactation.

**Findings**	1. **Identity markers**	2. **Bonding**	3. **Gender roles**	4. **Lack of knowledge**	5. **Lack of support**	**6. Predjudism**	**7. Resources**	8. **Sacrifice and inner conflict**
Cazorla-Ortiz G, Galbany-Estragués P, Obregón-Gutiérrez N, Goberna-Tricas J. Understanding the Challenges of Induction of Lactation and Relactation for Non-Gestating Spanish Mothers. J Hum Lact. 2020 Aug;36(3):528-536. doi: 10.1177/0890334419852939. Epub 2019 Jun 25. PMID: 31237797.		X		X		X		X
Falck FAOK, Dhejne CMU, Frisén LMM, Armuand GM. Subjective Experiences of Pregnancy, Delivery, and Nursing in Transgender Men and Non-Binary Individuals: A Qualitative Analysis of Gender and Mental	(X)			X	X	(X)		X

Health Concerns. Arch Sex Behav. 2024 May;53(5):1981-2002. doi: 10.1007/s10508-023-02787-0. Epub 2024 Jan 16. PMID: 38228983; PMCID: PMC11106200.								
Falck, F., Frisén, L., Dhejne, C., & Armuand, G. (2020). Undergoing pregnancy and childbirth as trans masculine in Sweden: Experiencing and dealing with structural discrimination, gender norms, and microaggressions in antenatal care, delivery, and gender clinics. International Journal of Transgender Health, 22(1-2), 42-53. https://doi.org/10.1080/26895269.2020.1845905	(X)			X	X		X	X
Galvão DLS, Araújo WJS, Brandão Neto W, Barros MBSC, Monteiro EMM. Intersectionality and challenges in support for chest-feeding transgender men. Cien Saude Colet. 2024 Apr;29(4):e19262023. Portuguese, English. doi: 10.1590/1413-81232024294.19262023. Epub 2023 Dec 15. PMID: 38655964.			X	X		X	X	X
Goldberg, A. E., Downing, J. B., & Sauck, C. C. (2008). Perceptions of children's parental preferences in lesbian two-mother households. Journal of Marriage and Family, 70(2), 419-434. https://doi.org/10.1111/j.1741-3737.2008.00491.x		X	X					
Hoffkling A, Obedin-Maliver J, Sevelius J. From erasure to opportunity: a qualitative study of the experiences of transgender men around pregnancy and recommendations for				X	X	X	(X)	

providers. BMC Pregnancy Childbirth. 2017 Nov 8;17(Suppl 2):332. doi: 10.1186/s12884-017-1491-5. PMID: 29143629; PMCID: PMC5688401.								
Jackson, J. E., Wild, R., Hallam, J., Graves, R., Woodstein, B. J., & Stothard, P. (2024). Exploring the healthcare experiences and support needs of chestfeeding or breastfeeding for trans and non-binary parents based in the United Kingdom. International Journal of Transgender Health, 25(4), 738–750. https://doi.org/10.1080/26895269.2023.2265371	X	X	X	X	X	X	X	
Juntereal NA, Spatz DL. Breastfeeding experiences of same-sex mothers. Birth. 2020 Mar;47(1):21-28. doi: 10.1111/birt.12470. Epub 2019 Nov 18. PMID: 31737946.		X		X	X	X	X	
Klittmark S, Niit JKP, Nerström E, Grundström H, Nieminen K, Wells MB, Malmquist A. LBTQ parents' needs for support postpartum following a complicated birth: A matter of reproductive justice. J Lesbian Stud. 2024;28(4):701-718. doi: 10.1080/10894160.2024.2367869. Epub 2024 Jul 25. PMID: 39049779.		(X)		X	X			
MacDonald T, Noel-Weiss J, West D, Walks M, Biener M, Kibbe A, Myler E. Transmasculine individuals' experiences with lactation, chestfeeding, and gender identity: a qualitative study. BMC Pregnancy Childbirth. 2016 May 16;16:106. doi: 10.1186/s12884-016-0907-y. PMID: 27183978; PMCID:	X	X		X	X	X	X	X

PMC4867534.								
Melo, F. E. B. and F. B. Scagliusi (2026). "Lactation induction, shared breastfeeding, and the right to opacity: a reflection on the use of health protocols and practices for women who have relationships with women." Einstein (Sao Paulo) 24(spe1): eRW2297. https://doi.org/10.31744/einstein_journal/2026RW2297		X			X	X	X	X
Pelka S. Sharing motherhood: maternal jealousy among lesbian co-mothers. J Homosex. 2009;56(2):195-217. doi: 10.1080/00918360802623164. PMID: 19197649.		X	X					
Pereira DMR, Peçanha LMB, Araújo EC, Sousa AR, Galvão DLS, Lima MFG, Souza MC, Torres KCC. Experiences and meanings of trans men about breastfeeding in light of the Theory of Social Representations. Rev Esc Enferm USP. 2024 Oct 21;58:e20240006. doi: 10.1590/1980-220X-REEUSP-2024-0006en. PMID: 39443286; PMCID: PMC11499245.		X	X	X		X		X
Rippey, P. L. F., & Falconi, L. (2017). A land of milk and honey? Breastfeeding and identity in lesbian families. Journal of GLBT Family Studies, 13(1), 16-39. https://doi.org/10.1080/1550428X.2015.1129297	X	X	X			X	X	X
van Amesfoort JE, van Rooij FB, Painter RC, Valkenburg-van den Berg AW, Kreukels BPC, Steensma TD, Huirne JAF, de Groot CJM, Van	X	X	(X)	X	X	(X)	(x)	

Mello NM. The barriers and needs of transgender men in pregnancy and childbirth: A qualitative interview study. Midwifery. 2023 May;120:103620. doi: 10.1016/j.midw.2023.103620. Epub 2023 Feb 16. PMID: 36893550.								
Ziegler, E., et al. (2026). ""They Didn't Know How to Support Me": The Experiences of Transgender Men and Non-Binary People With Chestfeeding." J Hum Lact 42(1): 94–104. https://doi.org/10.1177/08903344251401916	X	X	X	X	X	X	X	X
